# Long-Term Hyperphagia and Caloric Restriction Caused by Low- or High-Density Husbandry Have Differential Effects on Zebrafish Postembryonic Development, Somatic Growth, Fat Accumulation and Reproduction

**DOI:** 10.1371/journal.pone.0120776

**Published:** 2015-03-23

**Authors:** Sandra Leibold, Matthias Hammerschmidt

**Affiliations:** 1 Institute of Developmental Biology, University of Cologne, Cologne, Germany; 2 Cologne Excellence Cluster on Cellular Stress Responses in Aging-Associated Diseases (CECAD), University of Cologne, Cologne, Germany; 3 Center for Molecular Medicine Cologne (CMMC), University of Cologne, Cologne, Germany; University of North Carolina at Chapel Hill, UNITED STATES

## Abstract

In recent years, the zebrafish (Danio rerio) has emerged as an alternative vertebrate model for energy homeostasis and metabolic diseases, including obesity and anorexia. It has been shown that diet-induced obesity (DIO) in zebrafish shares multiple pathophysiological features with obesity in mammals. However, a systematic and comprehensive analysis of the different pathways of energy expenditure in obese and starved fish had been missing thus far. Here, we carry out long-term ad libitum feeding (hyperphagia) and caloric restriction studies induced by low- or high-density husbandry, respectively, to investigate the impact of caloric intake on the timing of scale formation, a crucial step of postembryonic development and metamorphosis, and on somatic growth, body weight, fat storage and female reproduction. We show that all of them are positively affected by increased caloric intake, that middle-aged fish develop severe DIO, and that the body mass index (BMI) displays a strict linear correlation with whole-body triglyceride levels in adult zebrafish. Interestingly, juvenile fish are largely resistant to DIO, while BMI and triglyceride values drop in aged fish, pointing to aging-associated anorexic effects. Histological analyses further indicate that increased fat storage in white adipose tissue involves both hyperplasia and hypertrophy of adipocytes. Furthermore, in ovaries, caloric intake primarily affects the rate of oocyte growth, rather than total oocyte numbers. Finally, comparing the different pathways of energy expenditure with each other, we demonstrate that they are differentially affected by caloric restriction / high-density husbandry. In juvenile fish, scale formation is prioritized over somatic growth, while in sexually mature adults, female reproduction is prioritized over somatic growth, and somatic growth over fat storage. Our data will serve as a template for future functional studies to dissect the neuroendocrine regulators of energy homeostasis mediating differential energy allocation.

## Introduction

Obesity, primarily defined as excessive or abnormal fat accumulation, is a major health problem. The World Health Organisation estimates that more than 500 million people (aged 20 years or older) were obese in 2008, more than 10% of adults worldwide and numbers are predicted to increase even further [1–3]. This is especially alarming given the association between obesity and diverse pathological conditions, including type 2 diabetes, hypertension, cardiovascular disease, hyperlipidemia, non-alcoholic fatty liver, gallstones, respiratory failure and certain types of cancer [4–7], which make obesity a leading risk factor for global mortality [8,9].

Obesity can be caused by genetic and environmental factors [10], leading to impaired energy homeostasis, the balance between energy intake and energy expenditure [9–11]. Energy intake depends on quantity and quality of consumed food. In humans, the increasing availability of high-caloric and high-fat diets is thought to be a main contributor to obesity [9,12–14]. This special (non-genetic) type of obesity is called nutritional, dietary or diet-induced obesity (DIO) and is studied in several animal models [15–18]. C57BL/6J mice develop obesity associated with several metabolic abnormalities when fed ad libitum with high-fat diet, but remain lean when fed ad libitum with standard chow [19,20]. Of note, obesity induced by high-fat diet is not due to hyperphagia. Animals do not consume more food per mg body weight [21–23], but even learn to compensate and eat less compared to mice on standard chow [24–26].

Assimilated energy can be expended in different ways. Energy needed to maintain essential vital functions (molecular and cellular) in basal state is called basal or standard metabolic rate and is the major component of energy expenditure [27,28]. Thermogenesis is another important component of mammalian energy expenditure [27,28]. But also physical activity due to foraging is a high priority way of energy expenditure [29,30]. When these primary demands of the organism have been satisfied, remaining energy can be allocated to further ways of energy expenditure like reproduction, body growth or non-foraging physical activity. In addition, energy can be stored as body fat to be used during times of food deprivation [29]. Neutral fat is mainly stored in adipocytes of the white adipose tissues, which in obese mammals expand accordingly, involving adipocyte hyperplasia (increase in cell numbers) and hypertrophy (increase in cell volume) [31–33]. Like in humans, dyslipidemia is quite common in obese mice [34,35], mainly seen by elevated levels of plasma triacylglycerols (triglycerides; TG) and low-density lipoprotein cholesterol, whereas levels of high-density lipoprotein cholesterol are decreased [36–38]. Beside increased fat mass [19,20,39,40], obese mice and humans exhibit a variety of phenotypes such as increased somatic growth [19,20,41,42], decreased heat production [39,43–45], compromised fertility [39,40,46,47] and decreased oxygen consumption [44,48–50], demonstrating the tight connection between the different pathways of energy expenditure.

In recent years, the zebrafish has emerged as an alternative vertebrate animal model for human physiology, including energy homeostasis and metabolic diseases [51,52]. Tissues and organs of zebrafish, including white adipose tissue, are similar to those of mammals in terms of function and structure [17,53–56]. Furthermore, neural and endocrine signals regulating energy homeostasis are conserved between zebrafish and mammals [17,56–60]. In addition, DIO in middle-aged zebrafish shares common pathophysiological pathways with obesity in mammals [17,61,62], and the zebrafish model has been used to identify additional regulators of energy homeostasis and potential drugs to treat obesity [55,61–63]. However, there are also crucial differences between zebrafish and mammals. For example, genetic interferences with the hypothalamic system regulating energy homeostasis in mammals has major effects on fat storage, but very moderate effects on somatic growth [41,64], whereas in zebrafish, it is vice versa [56,60,65]. Also the impact of the system on reproduction in mammals is quite low compared to fish [60,66].

In the aforementioned fish studies, analyses were usually restricted to one or two pathways of energy expenditure (e.g. somatic growth and fat storage / obesity [17,56,61,62], or somatic growth and reproduction [67]), and to specific developmental stages / ages of the fish. Furthermore, how differences in the reproductive potential are achieved at cellular and histological levels, was not addressed. Finally, apart from a reference in the frame of a postembryonic staging atlas [68], there are no reports on the effect of food intake / fish density on the developmental timing of metamorphosis in juvenile zebrafish.

Here, we carry out a systematic and comprehensive analysis of energy expenditure in zebrafish DIO and caloric restriction models on four different processes (developmental timing, somatic growth, fat storage and reproduction) and over an extended time span (2 weeks—18 months of age). We show that hyperphagia causes accelerated somatic growth and postembryonic development in juvenile fish. Obesity only developed after 2 months of age, eventually leading to a two-fold higher body mass index (BMI) and 3.5-fold increased relative whole body triglyceride (TG) levels, associated with hypertrophy and hyperplasia of adipocytes. In addition, ovarian weight in females was up to 2.6-fold increased, largely due to enhanced oocyte growth rates. Finally, we show that upon caloric restriction, the different pathways of energy expenditure are differentially affected, and discuss the biological sense of this phenomenon.

## Material and Methods

### Zebrafish maintenance

Zebrafish of the Ekkwill strain were raised and maintained at 28°C under a 14 h light / 10 h dark cycle and in tanks with continuous water exchange and recycling (AQUA SCHWARZ GmbH, Göttingen, Germany) [69]. The Ekkwill strain has been inbred in our laboratory for over 15 generations. Embryos were obtained by natural mating. For comparative analyses of aged-matched fish kept under differential husbandry conditions (see below), siblings deriving from the same mating of a single parental pair were used.

To provoke obesity, different high-fat diets were tested: murine high-fat diet (#C1057, Altromin, Lippe, Germany; fat content: 35.5%), a custom-designed fish high-fat diet sample (Bionic Nature, Dahn, Germany; fat content: 37%), and frozen and manually shredded waxworms (caterpillar larvae of honeycomb moth (Galleria mellonella); Top-Insect, Meulebeke, Belgium; fat content 57%) for adult zebrafish (> 3 months of age); egg yolk powder (#11551, Pati-Versand, Herzlake, Germany; fat content: 55%) and artificial plankton powder (Ocean Star International Inc., Snowville, USA; fat content: 39.4%) for juvenile zebrafish (1 to 3 months of age). However, after few meals in a row, the fish refused the diets.

To compare different means of caloric restriction, fish were raised in 2.5 liter (L) tanks under the following conditions: 1. high amounts of food per fish at low density (HF-LD): daily feeding of 3x 100 ml paramecia from 5–13 days post fertilization (dpf), and 3x 10 drops of artemia from 14 dpf onwards, 5 fish per tank; 2. low amounts of food per fish at low density (LF-LD): daily feeding of 3x 10 ml paramecia from 5–13 dpf, and 3x 1 drop of artemia from 14 dpf onwards (10% of food given in 1), 5 fish per tank (as in 1); 3. low amounts of food per fish at high density (LF-HD): daily feeding of 3x 100 ml paramecia from 5–13 dpf, and 3x 10 drops of artemia from 14 dpf onwards (as in 1; 10x more than in 2), 50 fish per tank (10x more than in 1 and 2).

For all other experiments, fish were raised in groups of 5 (high amounts of food, low density; HF-LD), 25 (normal amounts of food, normal density; NF-ND) or 50 fishes (low amounts of food, high density; LF-HD) in 2.5 L tanks until an age of 3 months. Afterwards fish were maintained in 12 L tanks. Fish were sorted for sex as soon as visible and maintained at a female:male ratio of 3:2. Fish were fed two to three times a day (Table 1) and each tank got the same amount of food necessary to assure survival of all individuals in the LF-HD tanks (50 fish), while in HF-LD tanks (5 fish), the food was not completely eaten up between the different feedings (ad libitum conditions). Fish between 5 and 14 dpf were fed with paramecia and artificial plankton powder, fish from 15 dpf to 2.5 months of age with artemia and artificial plankton powder, and fish older than 2.5 months with artemia, frozen food and flakes (Table 1).

**Table 1:** Feeding regime for zebrafish of different ages.

age	Mo - Fr			Sa, Su	
	9:30 am	1 am	5 pm	10 am	4 pm
5–14 days	paramecia		powder	paramecia	powder
15 days - 2.5 months	artemia	artemia	powder	artemia	powder
2.5 - 6 months	artemia	flakes	frozen food	artemia	flakes
6–18 months	artemia		frozen food	artemia	flakes

Compositions of the different diets were: artificial plankton powder (Ocean Szar International Inc., Snowville, USA): protein 44.5%, fat 39.4%, crude fiber 9.2%, ash 4.6%, moisture 2.2%; flakes (Tetra GmbH, Melle, Germany): protein 47%, fat 10%, crude fiber 3%, ash 11%, moisture 6%; frozen oxheart (Fimö Aquaristik, Bünde, Germany): protein 11.2%, fat 0.8%, crude fiber 2.7%, moisture 82.4%; frozen artemia (Fimö Aquaristik, Bünde, Germany): protein 5%, fat 1%, crude fiber 0.9%, ash 0.8%, and moisture 92%.

All zebrafish experiments were approved by the national animal care committee (LANUV Nordrhein-Westfalen; 8.87–50.10.31.08.130; 84–02.04.2012.A253) and the University of Cologne.

### Determination of body length and whole-body, ovarian and somatic weight

For determination of body length and body weight, fish were anesthetized in 0.13% Tricaine (w/v). Body length was measured from the anterior tip of the mouth to the base of the caudal fin (standard length, SL) using millimeter paper (to 0.5 mm) or ImageJ software (to 0.01 mm). For determination of body weight, anesthetized fish were dried on Whatman paper and measured to one decimal point in milligrams. 0.5, 1, 2 and 3 months old fish mostly were measured in pools, 5, 6.5, 9, 12 and 18 months old fish were measured individually (same number of males and females; at least 5 male and 5 female fish per condition).

For determination of testis or ovarian weight, fish were sacrificed after determination of whole body weight as described above. Afterwards, ovaries or testes were surgically removed and weighted. Weight was measured to one decimal point in milligrams and ovarian weights were deducted from whole-body weight to obtain female somatic body weight.

### Histology and analysis of scale mineralization, adipocytes, keratinocytes, skeletal muscle and oocytes

Vital alizarin red staining of living fish was performed as previously described [70] to visualize mineralized scales. Longitudinal rows of mineralized scales were counted.

For histological analyses, adult zebrafish were fixed and decalcified in Bouin`s solution at room temperature for 7 days, dehydrated in a graded series of alcohols, cleared in Roti-Histol (Carl Roth, Karlsruhe, Germany), and embedded in paraffin. Sections of 8–10 μm thickness were stained with hematoxylin and eosin (H&E), using standard protocols.

For quantification of subcutaneous adipocytes, H&E stained longitudinal sections of five levels were selected and numbers and total areas per side measured using ImageJ. Adipocyte cell size of both sides (left/right) in each section was calculated as area divided by cell number. This technique for adipocyte size determination was validated by comparing obtained values with mean size data determined by measurement of single adipocyte cell sizes using ImageJ (S1 Fig.). For each condition and sex, two fish and two sections per level were analyzed.

Thickness of skin was determined underneath the last scale of each section using ImageJ and done for one section of each level used for analysis of adipocytes. The size of keratinocytes was determined in the same region by measuring the size of 10 single keratinocytes using Image J.

For quantification of skeletal muscular body proportion, level 3 of the sections used for adipocyte quantification was analysed using ImageJ. Total area of the body was measured, excluding head, swim bladder and internal organs. Muscle-whole body proportions were calculated as area of muscles determined with the Treshold_Colour plugin for ImageJ divided by total area of the body in percent.

For quantification of oocytes, sections of four levels were selected and numbers of oocytes of different stages per side and size of secondary oocytes measured using ImageJ. For each condition three females were analyzed. Staging was done as described before [71,72]. Maximal size of secondary oocytes was calculated via the mean of the five biggest oocytes measured per level for all analyzed females per condition.

### Lipid analysis

For quantification of lipid content, fish were sacrificed and whole body weight was measured as described above. Fish were stored at -80°C until analysis. Lipid analysis was performed by Susanne Brodesser (CECAD lipidomics platform). Up to an age of 3 months, fish were analyzed in pools, older fish were analyzed individually (5 male individuals per condition).

Zebrafish samples were homogenized in water using the Homogenisator Precellys 24 (Peqlab, Erlangen, Germany) at 6.500 rpm for 30 sec. After lyophilization of the sample, the dry weight was determined. Lipids were extracted as previously described [73]. To determine lipid contents, lipid extracts were applied to 20 × 10 cm high performance thin layer chromatography (HPTLC) Silica Gel 60 plates (Merck, Darmstadt, Germany), which were pre-washed twice with chloroform/methanol 1:1 (v/v) and air-dried for 30 min. For the quantification of triacylglycerols each lane of the TLC plate was loaded with the equivalent of 30 μg zebrafish dry weight. The TLC solvent system used for detection of triacylglycerols was hexane/toluene 1:1 (v/v), followed by hexane/diethyl ether/glacial acetic acid 80:20:1 (v/v/v). For the quantification of cholesterol, the equivalent of 400 μg dry weight was applied to 20 × 20 cm TLC plates, which were developed in hexane/diethyl ether/formic acid 30:50:1 (v/v/v). Quantitative analytical TLC determination was performed as previously described [73].

### RNA extraction and quantitative real-time PCR

Total RNA was purified from whole fish with the PureLink RNA Mini Kit (Life Technologies, Carlsbad, USA) after homogenization of the frozen material by pestling in liquid nitrogen and RNA extraction with trizol (Invitrogen, CA, USA). cDNA was synthesized with Superscript II Reverse Transcriptase (Invitrogen, CA, USA) according to the manufactor`s instructions.

Quantitative real-time PCR (qRT-PCR) was performed in triplicates (3 experiments each) with an Applied Biosystems 7500 Fast Real-Time PCR system (Applied Biosystems, Foster City, USA) and TaqMan primer sets (Applied Biosystems, Foster City, USA), exploring of the following genes: *eef1a1l1* (eukaryotic translation elongation factor 1 alpha 1, like 1; Dr03432748_m1), *hmgcra* (3-hydroxy-3-methylglutaryl-coenzyme A reductase A; Dr03428716_m1) and *rps23* (ribosomal protein S23; Dr0343030371_m1). Relative mRNA expression levels of *hmgcra* were determined with Biosystems Prism SDS and Excel software, using the expression level of *eef1a1l1* or *rps23* as an internal standard.

### Statistics

Values are shown as mean ± standard deviation. Statistical significance among several experimental groups was determined by one-way analysis of variance (ANOVA), followed by Least Significant Difference (Bonferroni’s) test. Student’s *t*-test was used for comparisons between two groups. Significance was at p<0.05. Correlation analysis between BMI values TG levels was performed via least squares regression analysis and calculation of the Pearson product-moment correlation coefficient r.

## Results

In mice, strongest obesity is obtained upon feeding of high-fat diet. We offered different high-fat diets to zebrafish of different ages (for details see Material and Methods). However, these diets were usually refused after a few meals. Therefore, we had to apply an ad libitum feeding / hyperphagia versus caloric restriction approach, providing different amounts of regular chow. Consistent with previous analyses [17,56–59], supply of 10x more food led to significant higher linear growth rates of larval and juvenile zebrafish between 2 weeks and 3 months of age (S2 Fig.). An even more dramatic difference in growth rates was obtained when identical amounts of food were provided to correspondingly different numbers of fish per tank (S2 Fig.). However, fish kept at higher density did not show apparent signs of stress or altered feeding behaviour, consistent with former results [74,75]. In addition, standard deviations in the high-density group were not higher than those in the low-density group (S2 Fig.), suggesting that social factors, such as dominant and subordinate behaviours [76] have no stronger impact on food uptake under the chosen high-density conditions. Therefore, and because it is logistically easier especially when feeding diets other than paramecia and artemia, we applied the low density / high density approach for our long-term (2 weeks—18 months) experiments, raising and keeping fish in groups of 5 (high amounts of food per fish, low density; abbreviated as HF-LD), 25 (standard conditions; [69,77]; normal amounts of food per fish, normal density; NF-ND) or 50 (low amounts of food per fish, high density; LF-HD) individuals, while providing identical amounts of food per group.

### Caloric intake affects linear growth rates and developmental timing

**Fig 1:**
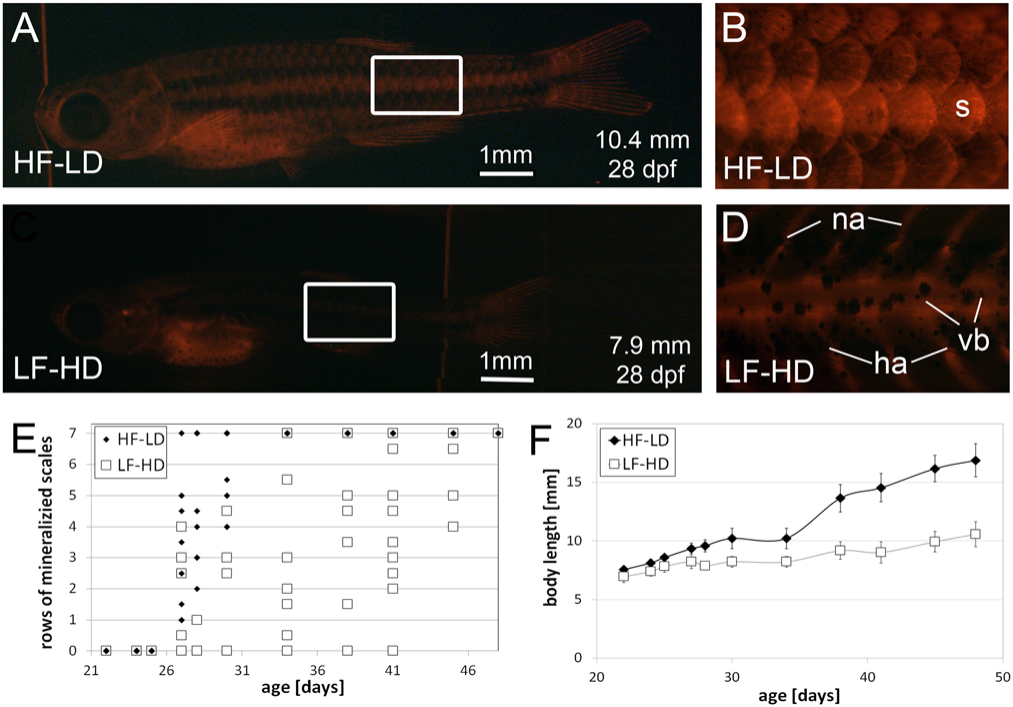
Hyperphagia results in increased linear growth and earlier scale formation A-D: Alizarin red staining of age-matched (28 dpf) juvenile fish (A,C) and corresponding magnification of the flank (B,D) of fish raised with high amounts of food at low density (A,B; HF-LD) and low amounts of food at high density (C,D; LF-HD). Body lengths of the shown individuals are indicated. Regions magnified in (B,D) are boxed in (A,C). E: Graph summarizing scale mineralization status (number of rows of mineralized scales) of analyzed fish at different ages. HF-LD fish started to display 7 rows of mineralized from an age of 27 days onwards, while it took LF-HD fish at least 34 days to reach this stage. F: Body length growth curves of sibling fish as in (A-E) shown as mean +/- standard deviation of 10 analyzed fish per condition and time point. LF-HD fish are significantly shorter than HF-LD fish. Similar results were obtained in a second, independent experiment. Abbreviations: ha, hemal arch; dpf, days post-fertilization; na, neural arch; s, scale; vb, vertebral body.

To investigate whether in addition to linear growth of the fish (Fig. 1F), other processes of postembryonic zebrafish development are affected by caloric intake, we analyzed the timing of scale formation. Scales are formed during metamorphosis, which under standard conditions occurs between the third and fifth week of development [78–80]. HF-LD fish raised at low density (excess of food) had formed seven longitudinal rows of scales at an age of 4.5 weeks, whereas LF-HD fish raised at high density (caloric restriction) took up to 6.5 weeks to reach this state (Fig. 1A-E). This indicates that in addition to somatic growth, caloric intake affects the timing of postembryonic developmental processes that occur during metamorphosis of the fish.

### Caloric intake affects body length, body weight and fat deposition

**Fig 2:**
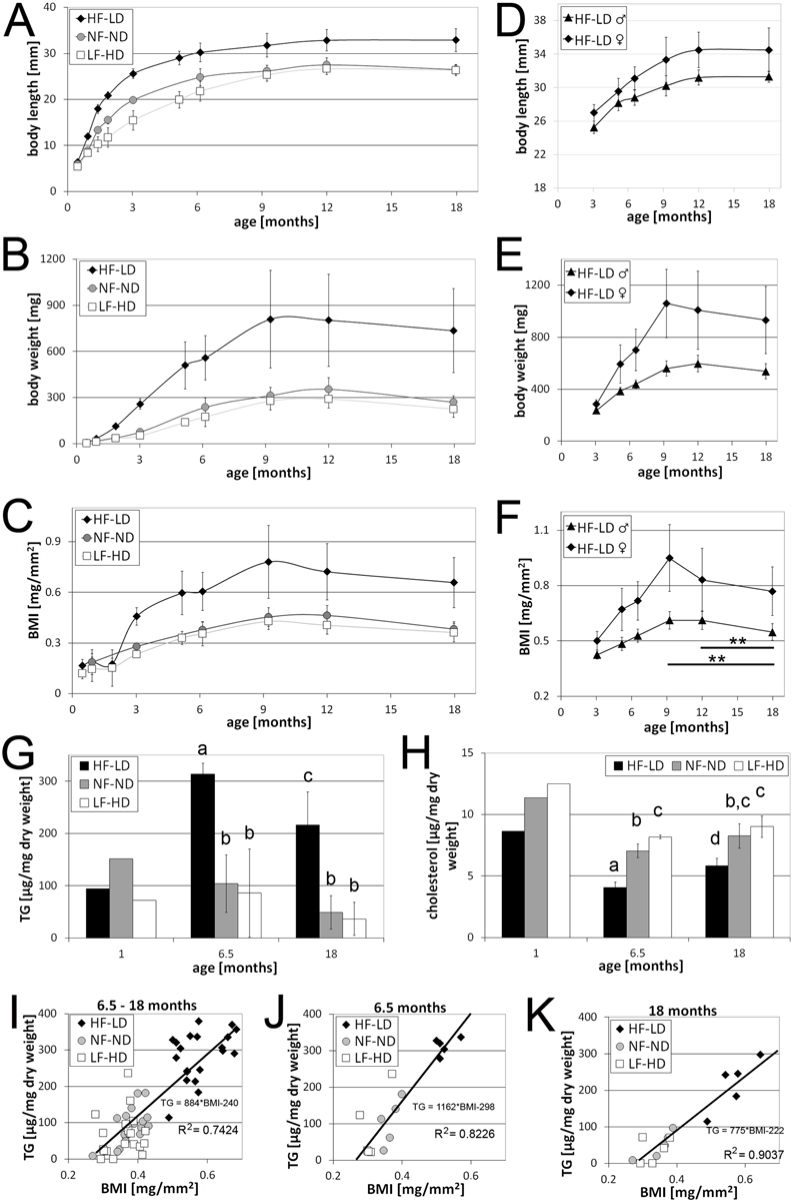
Hyperphagia results in increased body length, body weight, body mass index and triglyceride contents, but descreased cholesterol levels. A-F: Body length (A,D), body weight (B,E) and BMI (C,F) of LF-HD, NF-ND and HF-LD fish; (A-C) pooled data for males and females between 2 weeks and 18 months of age, n ≥ 10; same numbers of males and females analyzed; (D-F) separate evaluation of HF-LD males and HF-LD females between 3 and 18 months (for LF-HD and NF-ND fish, see S3A-F Fig.), ** indicates significant differences with p<0.01, calculated with ANOVA and the Least Significant Difference (Bonferroni’s) test; n ≥ 5. Similar results were obtained in two additional, independent experiments. G,H: Whole body triglyceride (TG) (G) and whole body cholesterol levels (H) of LF-HD, NF-ND and HF-LD at 1, 6.5 and 18 months of age (for more ages and curves, see S3G,H Fig.); columns with same superscript letter (a,b,c) are not significantly different (p>0.05) according to ANOVA followed by the Least Significant Difference (Bonferroni’s) test. For 1 months old fish, pools of 10 (HF-LD), 25 (NF-ND) or 50 fish (LF-HD) were analysed, while for older ages, at least 5 male individuals per condition were analysed (n ≥ 5). Similar results were obtained in an additional, independent experiment. I-K: Correlation between BMI and whole body TG for male LF-HD, NF-ND and HF-LD fish. (I) Pooled data for individual 6.5, 9, 12 and 18 months old fish; (J,K) Separate data for 6.5 months (J), and 18 months (K) old fish; for separate data for 9 and 12 months old fish, see S3K,L Fig.; the coefficient of determination (R^2^; square of correlation coefficient r) is higher when only fish of the same age are considered, as TG / BMI ratios progressively drop with the age of the fish. Perfect correlation: r = 1; R^2^ = 1.

We also carried out long-term experiments, keeping fish up to an age of 18 months (see Materials and Methods for age-specific feeding regimes). Growth performance of LF-HD, NF-ND and HF-LD fish was analyzed by determining body length (standard length, SL) and body weight and calculating the body mass index (BMI) at multiple time points from 14 days to 18 months of age. BMI was calculated by dividing the body weight (mg) by the square of the body length (mm). As adults, HF-LD fish showed significantly increased body lengths, body weights and BMIs compared to NF-ND or LF-HD fish (Fig. 2A-C).

Under all conditions (HF-LD, NF-ND, LF-HD), the increase of body length occurred according to a saturation curve, as characteristic for fish with determinate growth [81]. Growth rates were highest during the first months of age and progressively declined afterwards, with body lengths reaching a plateau at approximately 9 months of age. Growth rates of HF-LD fish were initially much higher than those of NF-ND and LF-HD fish, but also declined faster (Fig. 2A). Similar to body length, all fish (HF-LD, NF-ND, LF-HD) reached maximal body weights between 9 and 12 months. However, weight growth rates only started to decline much later than length growth rates, so that differences in body weights between HF-LD, NF-ND and LF-HD fish became progressively more pronounced than the differences in their lengths (Fig. 2B). For instance, between 3 and 6 months of age, HF-LD fish only grew 4.6 mm in length, less than the LF-HD fish (6.4 mm), whereas during the same time, HF-LD fish gained 300 mg body weight, compared to 123 mg of LF-HD fish, pointing to an increase in the “thickness” of HF-LD fish. Interestingly, while body length remained constant after 12 months of age, body weights of all fish (HF-LD, NF-ND, LF-HD) dropped between 12 and 18 months, pointing to some kind of aging-associated “thinning”. These features of length and weight growth were also reflected by the BMI (Fig. 2C). During the first 2 months, BMI values were rather constant (0.16 mg/mm^2^), and differences between HF-LD and NF-ND or LF-HD fish were minor. However, BMI values continuously increased afterwards, reaching maximal values at 9 months, with significant differences between HF-LD (0.78 mg/mm^2^) and NF-ND (0.45 mg/mm^2^) or LF-HD fish (0.42 mg/mm^2^), while during further aging (12 and 18 months), values dropped again for all groups.

Of note, weight and BMI values of individual fish of each group were quite variable, as reflected by the rather high standard deviations in Fig. 2B,C. To investigate to which extent this is due to differences between males and females—in sexually mature females ovaries can account for up to 29% of the total body weight (see below), compared to 2% for the testis in male siblings (1.22 +/- 0.20% for LF-HD, 1.85 +/- 0.24% for HF-LD males at 9 months of age)—we also generated sex-specific body length, body weight and BMI curves, starting at an age of 3–6 months, when males and females could be macroscopically distinguished (Fig. 2D-F; S3A-F Fig.). Indeed, female fish were longer, heavier and had a higher BMI than their male siblings. In addition, they displayed more pronounced age-dependent shifts in body weights and BMIs (Fig. 2E,F). Generally, all sex-specific differences were stronger between HF-LD fish (Fig. 2D-F) than between NF-ND or LF-HD fish (S3A-F Fig.). However, while standard deviations were much lower for the male HF-LD subgroup, they remained comparably high for the female subgroup (Fig. 2D-F). Because of this high variability, the age-dependent decline in BMI values was only statistically significant for males, but not for females (Fig. 2F; S3C,F Fig.). This high variability seen in females is at least not soley due to differences in ovary dimensions, as standard deviations of somatic BMIs (after surgical removal of the ovaries) were only slightly lower (12.3%) than for whole body BMIs (15.3%), but still higher than for whole-body BMIs of sibling HF-LD males (8.1%), pointing to additional sex-specific variations in somatic traits.

In mammals, BMI is used as an indicator of anorexia and obesity. However, in addition to body fat, the BMI is also influenced by other factors, such as the relative amount of muscle tissue and, at least in females of non-amniotic vertebrates, the size of the ovaries (see above). To avoid the latter factor, we restricted the following lipid analyses to male fish only, comparing fat contents of HF-LD, NF-ND and LF-HD males of different ages. The main storage form of neutral lipids are triacylglycerols (triglycerides; TG). Determination of relative TG contents (in μg / mg dry weight) from whole fish revealed a strong dependence on age and feeding conditions of the fish (Fig. 2G; S3G Fig.). In general, HF-LD fish had significantly higher whole-body TG levels than NF-ND and LF-HD fish. However, increased TG levels were first visible from an age of 3 months onwards, whereas younger HF-LD fish seemed to have the same or even lower TG levels than LF-HD or NF-ND fish. Furthermore, TG levels of all fish (HF-LD, NF-ND and LF-HD) fish started to decline again between 9 and 12 months of age, pointing to aging-associated TG wasting (S3G Fig.). These shifts are in line with the BMI dynamics described above (Fig. 2C). Indeed, a strong linear correlation between whole-body TG concentrations and BMI values could be seen for adult HF-LD/NF-ND/LF-HD males (Fig. 2I; r = 0.8494, p<0.001, age 6 to 18 months), making the BMI a reliable indicator of obesity and anorexia. However, TG / BMI ratios continuously declined with age, as reflected by the different slopes of the regression lines, and the higher coefficients of determination (R^2^) of age-specific (Fig. 2J,K; S3K,L Fig.) compared to pooled data (Fig. 2I). Thus, in an older fish, a given BMI translates to a lower TG value than in a younger fish (compare Fig. 2J, Fig. 2K, S3K Fig. and S3L Fig. for 6.5, 9, 12 and 18 months old males). This points to an increasing impact of other, non-adipose tissues on the BMI of aging fish. As one possible factor, we compared muscle dimensions in H&E sections of middle-aged (6.5 months) and aged (18 months) HF-LD and LF-HD males. Indeed, in contrast to the lower TG levels (S3G Fig.), aged fish displayed rather stable muscle—whole body volume ratios (S3I Fig.). Accordingly, ratios between TG levels and muscle proportions in aged fish were 0.8 fold (HF-LD) or 0.5 (LF-HD) lower than in the corresponding middle-aged fish.

In addition to TG, we analyzed whole-body levels of cholesterol, another neutral lipid. In human, cholesterol is associated with obesity and can be found in high levels in the blood of obese individuals [82]. Interestingly, in zebrafish, whole-body cholesterol displayed responses almost opposite to those of TG (Fig. 2H and S3H Fig.). Thus, HF-LD fish contained lower cholesterol levels than LF-HD fish. Furthermore, cholesterol levels were highest in juveniles, continuously decreased up to an age of 9 to 12 months, and increased again thereafter. Cholesterol levels are influenced by cholesterol uptake via food, excretion and by endogenous cholesterol synthesis [65,83,84]. In obese humans and mice, cholesterol levels are high due to enhanced synthesis rather than increased cholesterol uptake or decreased excretion [85,86]. As a potential marker for cholesterol synthesis, we analyzed transcript levels of *hmgcra*, encoding 3-hydroxy-3-methylglutaryl-coenzyme A reductase A, the rate-limiting enzyme of cholesterol synthesis [87]. In young (1 month old), DIO-resistant HF-LD zebrafish, *hmgcra* mRNA levels were approximately 3-fold lower than in middle-aged (7 months old) DIO HF-LD fish (S3J Fig.), although whole-body cholesterol levels were about double as high (S3H Fig.). This suggests that the altered *hmgcra* mRNA levels are a secondary and compensatory consequence [88], rather than the cause, of the different cholesterol levels, while the mechanisms underlying the age-dependent shifts in cholesterol contents remain elusive (see Discussion).

### Caloric intake affects numbers and sizes of adipocytes

**Fig 3:**
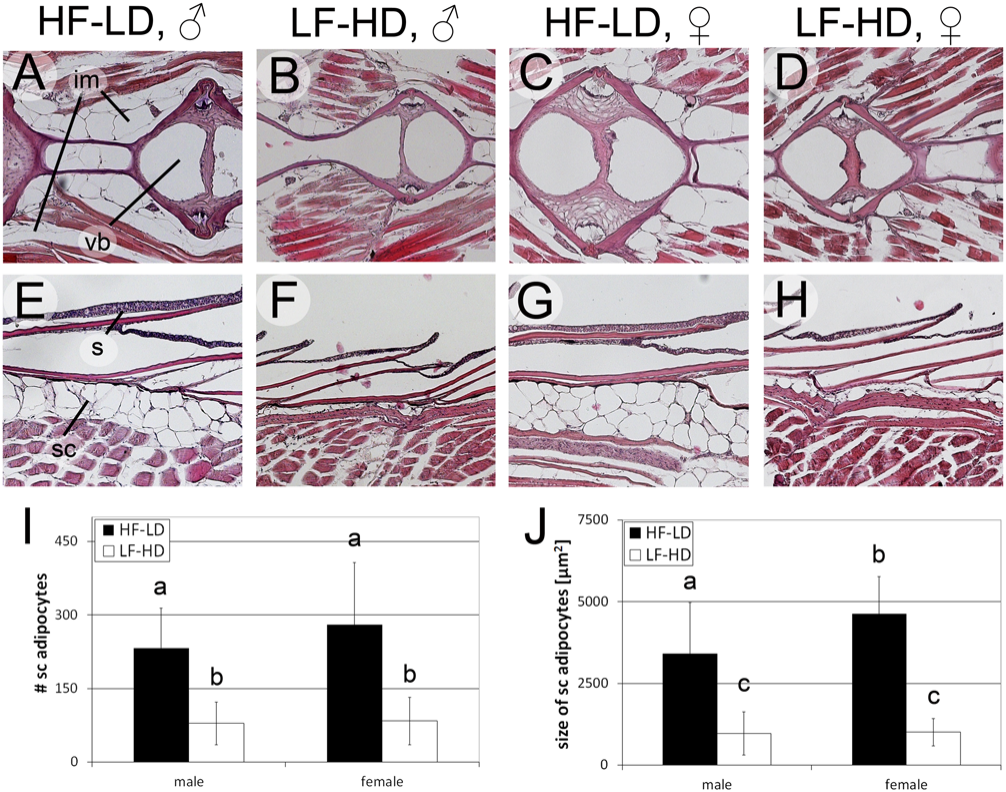
Hyperphagia results in hyperplastic and hypertrophic adipocytes in middle-aged fish. A-H: H&E staining of longitudinal sections of male (A,B,E,F) and female (C,D,G,H) HF-LD (A,C,E,G) and LF-HD (B,D,F,H) fish, 6.5 months of age, dorsoventral level 2 (compare with S4A Fig.); I-J: Average total numbers of subcutaneous adipocytes per level (I) and sizes (J) of subcutaneous adipocytes of male and female LF-HD and HF-LD fish (body length 28.8 +/- 0.9 mm (male HF-LD), 21.2 +/- 1.5 mm (male LF-HD), 31.1 +/- 1.4 mm (female HF-LD) and 20.8 +/- 1.4 mm (female LF-HD)) from 5 corresponding longitudinal levels along the dorsoventral axis (see S4C,D Fig.); columns with different superscript letters are significantly different (p<0.05) according to ANOVA followed by the Least Significant Difference (Bonferroni’s) test, n = 40 (2 fish per condition, 5 levels per fish, 2 sections per level and left and right sides per section). Similar experiments were obtained in an additional, independent experiment. Abbreviations: im, intermuscular adipocytes; s, scale; sc, subcutaneous adipocytes; vb, vertebral body.

In mammals, adipose tissue is the major site of TG and cholesterol storage [89,90]. As in mammals, zebrafish fat is stored as lipid droplets in adipocytes within regionally distinct white adipose tissues in subcutaneous (sc), visceral (vs) and intermuscular (im) locations [17,53,54,56]. Obese mammals exhibit more and larger adipocytes than lean siblings [31–33,91]. To study whether this also applies to DIO in zebrafish and whether aging influences adipocytes, we compared adipocyte numbers and sizes in HF-LD and LF-HD fish of two different ages. At an age of 6.5 months, shortly before maximal differences in the BMI and TG values were obtained (see above), H&E stained longitudinal sections revealed more and larger adipocytes in visceral, intermuscular (Fig. 3A-D) and subcutaneous (Fig. 3E-H) regions of HF-LD fish. This was confirmed by quantitative analyses of subcutaneous adipocytes, evaluating sections at 5 different levels along the dorsoventral body axis of the fish (S4A Fig.). Of note, adipose tissue dimensions and their responses to different husbandry conditions varied along the dorsoventral axis of the fish. Thus, LF-HD fish displayed highest subcutaneous adipocyte numbers and sizes at level 2 (dorsal), HF-LD males highest adipocyte numbers at level 2, but highest adipocyte sizes at level 5 (ventral) (S4C,D Fig.). This indicates that caloric intake has differential and region-specific hyperplastic and hypertropic effects on subcutaneous adipocytes, and that ventral adipocytes are most susceptible to diet-induced hypertrophy. In average, HF-LD fish possessed approximately 3x more (p<0.001 for males and females) and about 4x larger (p<0.001 for males and females) subcutaneous adipocytes than LF-HD fish (Fig. 3I-J). Furthermore, in particular the effect on adipocyte size was more pronounced in HF-LD females than in HF-LD males (p<0.001 for size; p = 0.14 for number), whereas no significant sex-specific differences in adipocyte numbers and sizes could be observed for the lean (LF-HD) siblings (p>0.2 for size and number). In contrast to adipocytes, thickness of skin and size of keratinocytes were not altered between male LF-HD and HF-LD fish, while LF-HD females possessed an even thicker skin and only slightly smaller keratinocytes than HF-LD females (S4E,F Fig.). In conclusion, DIO was tightly associated with adipocyte hyperplasia and hypertrophy, whereas the numbers and sizes of other cell types were hardly affected, suggesting that hyperphagia specifically promotes both the proliferation and growth of adipocytes. Of note, similar shifts were observed when comparing adipocytes of size-matched (rather than age-matched) HF-LD and LF-HD fish (see below), indicating that adipocyte hypertrophy and DIO of HF-LD fish are at least not solely caused by their higher growth rate and larger body size, but by the actual husbandry conditions (hyperphagia at lower density).

**Fig 4:**
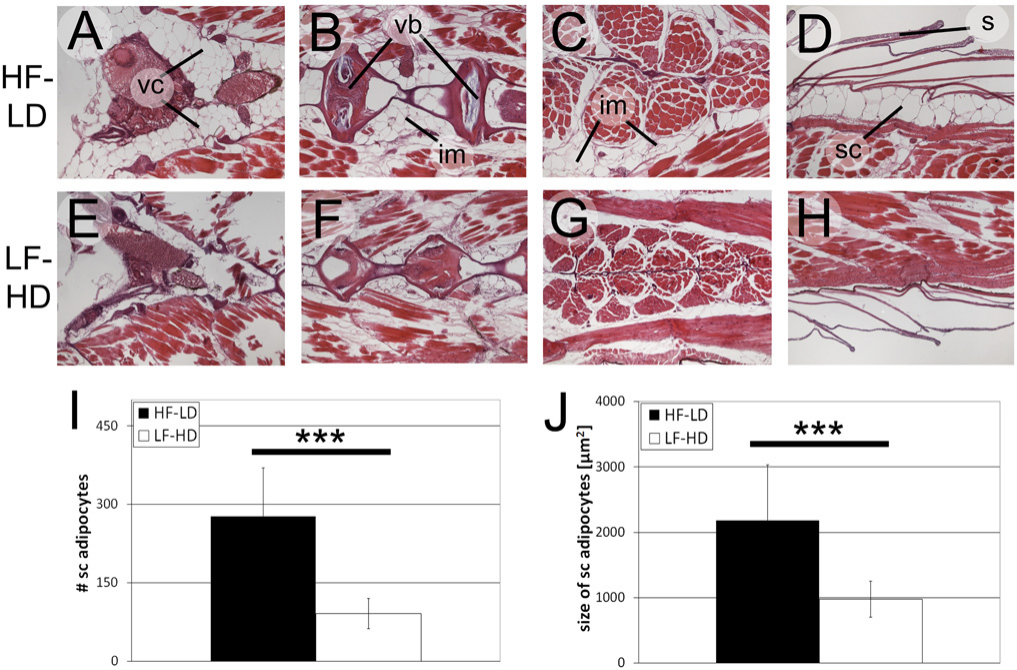
Aged (18 months old) male HF-LD and LF-HD fish display decreased adipocyte sizes but unaltered adipocyte numbers compared to middle-aged fish. A-H: H&E staining on longitudinal sections of male LF-HD (E-H) and HF-LD (A-D) fish showing visceral (A,E), subcutaneous (D,H) and intermuscular (B,C,F,G) adipocytes at dorsoventral levels 2 (A,B,D,E,F,H) or level 4 (C,G) (compare with S5A Fig.); I,J: Average total numbers of subcutaneous adipocytes per level (I) and sizes (J) of subcutaneous adipocytes of male LF-HD and HF-LD fish (body length 32.3 +/- 1.0 mm (male HF-LD), 22.5 +/- 1.0 mm (male LF-HD)) from 5 corresponding longitudinal levels along the dorsoventral axis (S5C,D Fig.); *** indicates significant differences (p<0.001) according to the Student`s T test, n = 40 (2 fish per condition, 5 levels per fish, 2 sections per level and left and right per section). Similar results were obtained in an additional, independent experiment. Abbreviations: im, intermuscular adipocytes; s, scale; sc, subcutaneous adipocytes; vb, vertebral body; vc, visceral adipocytes.

In light of the observed decline of BMI and TG content values in aged fish (see above; Fig. 2), we also examined adipocytes in 18 months old male LF-HD and HF-LD fish (Fig. 4 and S5 Fig.). As at 6.5 months of age, HF-LD fish had significantly more and larger visceral, intermuscular and subcutaneous adipocytes than LF-HD fish (Fig. 4A-J), whereas differences in the numbers and sizes of keratinocytes were minor (S5E,F Fig.). Interestingly, while numbers of subcutaneous adipocytes in 6.5 and 18 months old fish were similar or even slightly increased in aged fish (80 / 90 for LF-HD fish, p>0.05; 230 / 280 for HF-LD fish, p<0.05; compare Fig. 3I with Fig. 4I), adipocyte sizes in aged HF-LD fish were significantly smaller than in the 6.5 months old HF-LD fish (3400 / 2200 μm^2^, p<0.001; compare Fig. 3J with Fig. 4J) and unchanged in LF-HD males (970 / 980 μm^2^, p>0.05; compare Fig. 3J with Fig. 4J). This size reduction was particularly prominent in ventral regions of HF-LD fish (level 5: 5300 / 2600; compare S4D Fig. with S5D Fig.), thus, the regions that contained the largest subcutaneous adipocytes in 6.5 months old HF-LD fish (see above), whereas more dorsal regions were less affected. Together, this indicates that the “thinning” of aged fish is largely due to aging-associated and region-specific shrinkages of adipocytes.

### Caloric intake affects female reproduction

**Fig 5:**
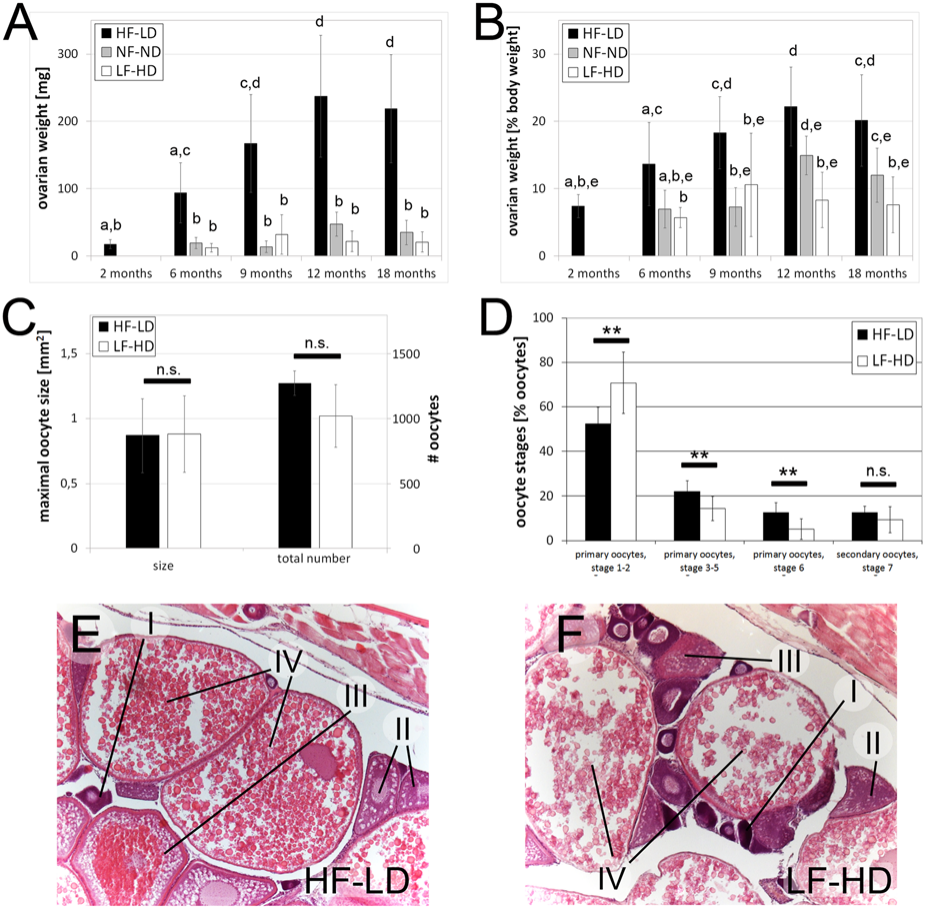
Hyperphagia leads to increased ovarian sizes and enhanced oocyte growth rates, while not affecting final oocyte sizes. A-B: Weight of ovaries of female LF-HD, NF-ND and HF-HD in mg (A) and in % total body weight (B) at different ages; at an age of 2 months, numbers could only be supplied for HF-LD females, since NF-ND and LF-HD fish of this age were not sexually mature as yet; columns with same superscript letter are not significantly different (p>0.05) according to ANOVA followed by the Least Significant Difference (Bonferroni’s) test, n ≥ 5. C: Sizes of fully grown oocytes and total number of oocytes of female LF-HD and HF-LD fish were not significantly (n.s.) different according to the Student`s T test, n = 10 (10 biggest oocytes of all levels) or n = 3 (3 females). D: Relative numbers of oocytes per maturation stage of HF-LD and LF-HD females (in % of total oocyte numbers) in all of the four analyzed dorsoventral levels (compare with S6A Fig.); ** indicates significant differences (p<0.01), n.s. means not significant according to the Student`s T test, n = 3. E-F: H&E staining of longitudinal sections through ovaries of HF-LD (E) and LF-HD female (F), 8.5 months of age, dorsoventral level 2. I: primary oocyte, stage 1–2 (previtellogenic); II: primary oocyte, stage 3–5 (previtellogenic); III: primary oocyte, stage 6 (vitellogenic); IV: secondary oocyte (meiotic arrest), stage 7; staging was done as described [71,72]. Similar results were obtained in second, independent experiments.

As described above (Fig. 2D-F), HF-LD females displayed much more pronounced age-dependent shifts in body weight and BMI than sibling males. These shifts might be partly due to the acquirement and aging-associated loss of reproductive activity. To look into this more directly, and to study the effect of caloric intake on female reproduction, we analyzed the weights of ovaries and oocyte sizes and numbers in HF-LD, NF-ND and LF-HD females of different ages. In all conditions (HF-LD, NF-ND, LF-HD), absolute (in mg; Fig. 5A) and relative (in % of total body weight; Fig. 5B) ovarian weights increased between 3 and 12 months of age, whereas they slightly decreased again between 12 and 18 months of age. Furthermore, at all investigated ages, ovaries of HF-LD fish were significantly larger / heavier than in LF-HD fish, accounting for approximately 22% of the total body in 12 months old HF-LD fish, compared to 8% in LF-HD fish. This indicates that caloric intake affects female reproduction. At first sight it even seems to suggest that food intake has a stronger impact on oogenesis than on somatic growth. However, this is misleading, as HF-LD females were larger and heavier than their age-matched LF-HD siblings. Therefore, LF-HD fish also need to be compared with size-matched, and thus much younger, but already sexually mature HF-LD females. For this purpose, we also determined ovarian weights of 2 months old HF-LD females, which were of about the same body weight as 6 months old LF-HD females. There was no significant difference in absolute or relative ovarian weights observed between those fish (Fig. 5A,B, left columns; see also below).

We also analyzed ovarian growth at cellular level. Ovaries of sexually mature females are composed of oocytes of different stages. After enveloping by pre-follicle cells, oocytes start to increase in size and weight, largely due to vitellogenesis, the progressive incorporation of yolk produced in the liver [72,92,93]. Analysis of oocytes in longitudinal sections of sexually mature, 6.5 and 8.5 months old females at different dorsoventral levels (S6A,B Fig.) revealed comparable maximal sizes of fully grown oocytes in LF-HD and HF-LD females (Fig. 5C; p = 0.64). Also, the total number of oocytes in HF-LD fish was only moderately increased (p = 0.15), which most likely is a reflection of their increased overall body size compared to LF-HD fish. However, there were pronounced differences in the distribution of oocyte sizes, with HF-LD females possessing relatively fewer oocytes of previtellogenic stages 1–2, but significantly more of the more advanced stages 3–7 (Fig. 5D-F; see S6D Fig. for total numbers). Together, this indicates that ovaries primarily grow due to enhanced oocyte growth and maturation rates, rather than increased numbers or final sizes of mature oocytes.

### Upon caloric restriction, scale formation is prioritized over somatic growth

**Fig 6:**
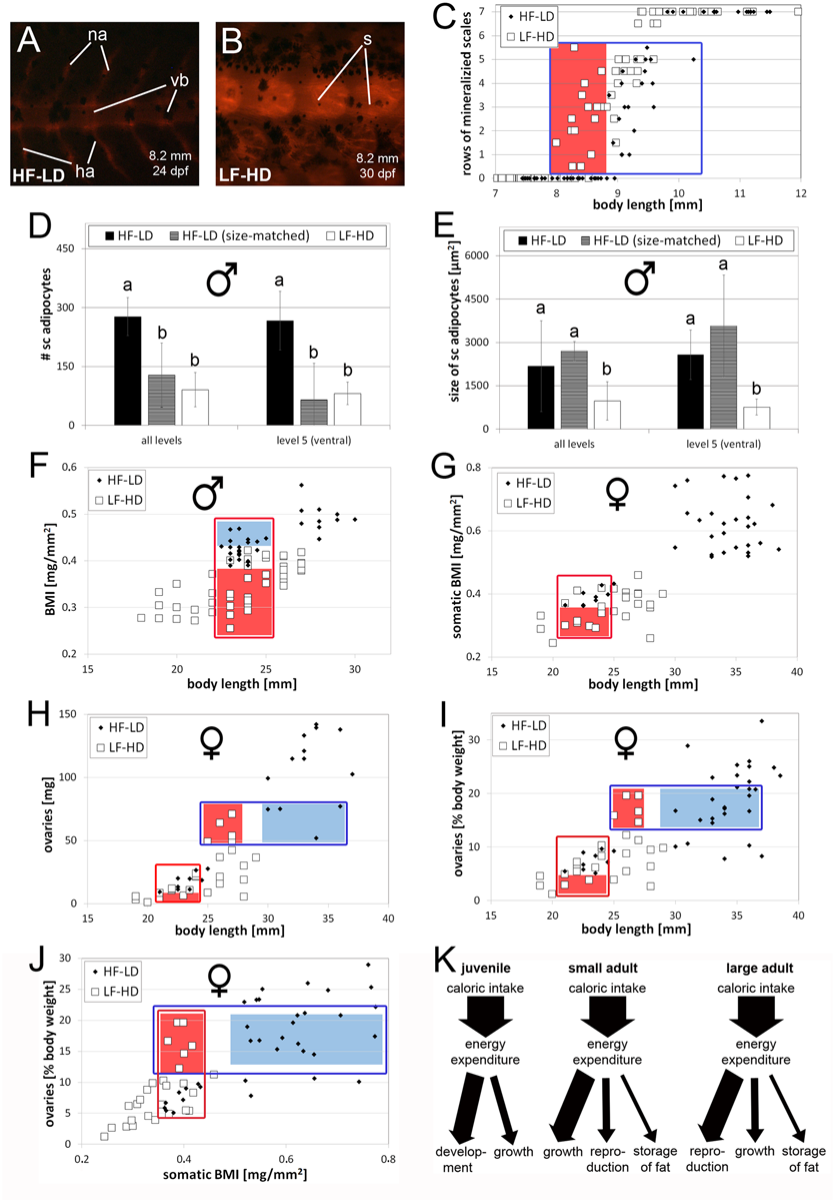
Caloric intake has differential effects on the timing of scale formation, somatic and ovarian growth and fat incorporation. A-B: Alizarin red staining of size-matched juvenile HF-LD (A) and LF-HD (B); body lengths and ages of the shown individuals are indicated. The flank of the LF-HD fish has several rows of mineralized scales, whereas no scales are visible in the corresponding region of the HF-LD fish. (C) Plot of rows of mineralized scales vs body length. (D,E) Comparison of numbers (D) and sizes (E) of subcutaneous adipocyte sizes (E) of 18 months old LF-HD males (white columns) with age-matched HF-LD male siblings (black column) and size-matched HF-LD males (striped columns; age: 2 months). Standard lengths of investigated fish were: HD-LD, 18 months: 32.3 +/- 1.0 mm; HF-LD, 2 months: 23.0 +/- 0,8 mm; LF-HD, 18 months; 22.5 +/- 1.0 mm. Left sides of each panel show average numbers from 5 corresponding longitudinal levels along the entire dorsoventral axis (for averages of single levels, see S7 Fig.), right sides show numbers for level 5 only (ventral). Columns with different superscript letter are significantly different (p<0.05) according to ANOVA followed by the Least Significant Difference (Bonferroni’s) test; n = 40 (2 fish per condition, 5 levels per fish, 2 sections per level and left and right side per section). F-J: Plots of BMI vs length of males (F); somatic BMI vs. length of females (G); absolute ovary weight vs. length (including 2 months HF females) (H); relative ovary weight vs. length (I); relative ovarian weight vs. somatic BMI (J). Crucial parameter intervals in (C, F-J) are boxed in red or blue, intervals only occupied by HF-LD fish are highlighted in blue, intervals only occupied by LF-HD fish in red. In (G-J), HF-LD females in regions boxed in red were 2 months of age, but already sexually mature. K: Schemes showing differential energy allocations to scale formation / postembryonic development, growth, reproduction and fat storage in juvenile, small and large adult fish. Abbreviations: ha, hemal arch; na, neural arch; s, scale; vb, vertebral body.

Thus far, the different caloric intake-dependent processes (scale formation, somatic growth, fat accumulation, female reproduction) have been evaluated as a function of the age of the fish. To study whether they are differentially affected, we also compared them with each other. Plotting the extent of scale formation (postembryonic development) of juvenile HF-LD and LF-HD fish (3–6.5 weeks of age; Fig. 1) as a function of their body length, it became apparent that compared to HF-LD fish, LF-HD fish initiated scale formation at smaller body sizes (Fig. 6A-C). This indicates that upon caloric restriction in juvenile fish, at least some processes of metamorphosis are prioritized over somatic growth (Fig. 6K, left panel).

### Upon caloric restriction, reproduction and somatic growth are prioritized over fat storage

Differential responses to caloric intake were also observed for the processes analyzed in older fish (2–18 months; Figs. 2, 3, 4, and 5). Comparisons of subcutaneous adipose tissue revealed that 18 months old LF-HD males in average possessed adipocytes of similar numbers but smaller sizes than the faster-growing and correspondingly younger size-matched hyperphagic HF-LD fish (Fig. 6D,E). Differences were even more pronounced on the ventral side of the fish (Fig. 6D,E; S7A,B Fig.), where adipocytes are particularly susceptible to diet-induced hypertrophy (see above; S4 Fig.). In contrast, differences between 6.5 months old LF-HD fish and size-matched HF-LD fish were more prominent in dorsal levels, where the more slowly growing and older LF-HD fish displayed both fewer and smaller adipocytes than their size-matched HF-LD counterparts (S7 Fig.; level 1). This indicates that upon caloric restriction, energy is preferentially used for somatic growth rather than fat storage (Fig. 6K). The same results were obtained when plotting the BMI values against body lengths, revealing that LF-HD fish were generally leaner (lower BMI) than HF-LD fish of the same body length (Fig. 6F,G). However, differences were more pronounced in males (Fig. 6F) than in females (somatic BMI; Fig. 6G), possibly because energy dedicated to somatic growth in males also needs to go into reproduction in females (Fig. 6K). In line with this notion, plots of ovarian weights as a function of body length revealed differential effects of caloric intake on ovarian versus somatic growth, which even varied depending on the actual size of the females. At comparable smaller sizes, LF-HD females displayed slightly lower ovarian weights than HF-LD females (Fig. 6H,I; red boxes). However, at more advanced sizes, LF-HD females were smaller than HF-LD females when reaching comparable ovarian weights (Fig. 6H,I; blue boxes). This suggests that upon caloric restriction, energy is preferentially used for somatic rather than for ovarian growth when females are still small (Fig. 6K, middle panel), whereas energy is preferentially used for ovarian growth when they are larger (Fig. 6K, right panel). When plotting ovarian weights as a function of somatic BMI, ovarian weights of LF-HD females were higher than in HF-LD females with same BMI (Fig. 6J, red box). Consistently, LF-HD females were leaner (lower somatic BMI) than HF-LD females when reaching comparable ovarian weights (Fig. 6J, blue box), indicating that energy is preferentially used for ovarian growth, rather than fat storage.

In sum, this suggests that under caloric restriction, energy usage for fat storage is generally underrepresented compared to somatic and ovarian growth, while ovarian growth only becomes prioritized over somatic growth once females have reached a crucial body size (Fig. 6K; see also Discussion).

## Discussion

Thus far, a systematic and comprehensive analysis of the effects of differential caloric intake on the different pathways of energy expenditure in zebrafish had been lacking. Oka et al. (2010) [17] nicely demonstrated that diet-induced obesity (DIO) is characterized by accelerated somatic growth and increased body weight / TG levels, associated with pathophysiological molecular conditions similar to mammalian obesity. However, analyses were restricted to a rather short time span, feeding young adults different amounts of artemia for just 8 weeks and not considering possible effects on reproduction. Here, we included this important pathway of energy expenditure, and extended the analyses to younger and older ages, basically covering the entire life span of the fish and also addressing known human conditions such as juvenile obesity [94] or age-related weight loss / anorexia / cachexia [95–97]. Carrying out such long-term experiments, we also had to consider a fourth aspect of caloric intake. Thus, it is not only known to affect aging and life span [98], but also, although less studied, the developmental timing of sexual maturation [99] and possibly other processes of postembryonic development [68,80]. Therefore, we also studied the timing of scale formation, one of the processes of metamorphosis, which under standard conditions takes place between 3 and 5 weeks of development and which, in addition to scale formation and mineralization studied here, includes sex differentiation and the acquirement of the adult body pigmentation pattern and of the adult fins [68,79,100].

Since we wanted to keep the fish under hyperphagia and caloric restriction conditions throughout the entire time from 5 dpf, when they start to take up external food, through an advanced age of 18 months, we had to apply different age-specific feeding regimes. Therefore, rather than feeding different amounts of the diets to the same number of fish [17], as only done in a comparative pioneer experiment up to three months of age (S2 Fig.), we switched to a low density / high-density approach, as described formerly [101], providing identical amounts of food to tanks with 5 (HF-LD), 25 (NF-ND) or 50 (LF-HD) fish. This approach, while logistically much easier, resulted in more pronounced differences between LF-HD and HF-LD fish than the classical approach (S2 Fig.). Of course, we cannot rule out that in addition to reduced caloric intake, other factors caused by fish crowding have influenced the performance of the LF-HD fish. However, if so, these effects should have been minor, as the LF-HD groups were kept at densities of 4 fish/L, thus, only twice as high as in the commonly applied standard conditions (2 fish/L) [69,77], and 10–15x lower than the conditions in which crowding-induced increases in whole-body cortisol levels (40 fish/L) [74] or negative impacts on breeding efficiencies (60 fish/L) [102] had been formerly reported. Indeed, we could not detect any obvious changes in the feeding and social behaviors of LF-HD compared to NF-ND fish kept in parallel under standard conditions. Also, in all assays, LF-HD and NF-ND fish gave rather similar results compared to the much more pronounced differences between LF-HD and HF-LD fish. Furthermore, if LF-HD had been majorly affected by more adverse factors in addition to caloric restriction, we would have expected higher standard deviations compared to HF-LD fish, which, however, was not the case. That standard deviations were overall rather high could be due to genetic variability among individual fish, which were not isogenic. Yet, to keep such genetic disturbances at a minimum, we used a line that had been systematically inbred in our laboratory for over 15 generations. Furthermore, for the different age-matched LF-HD versus HF-LD experiments, only sibling fish from the same parental pair were used.

### Juvenile HF-LD fish do not develop DIO, but display earlier scale formation and increased somatic growth

In contrast to older fish, juvenile HF-LD fish up to an age of two months did not develop DIO, as reflected by almost identical BMI values (Fig. 2C) and TG levels (Fig. 2G) compared to NF-ND and LF-HD fish. Instead, excessive caloric energy was invested in faster somatic growth (Fig. 2A). This indicates that postembryonic growth rates do not only depend on the incubation temperature, but also on the feeding and husbandry conditons [68]. Of note, this DIO-resistant period corresponds to the phase during which growth rates are highest (Fig. 2A). In this light, it would be interesting to carry out similar studies in related fish species with indefinitive growth and constant linear growth throughout life [81], investigating whether they are also less susceptible to DIO during adulthood. Another factor possibly accounting for the resistance of juvenile zebrafish to DIO is metamorphosis, which takes place during this period and which also requires energy. Accordingly, scale formation in HF-LD fish took place significantly earlier than in LF-HD fish (Fig. 1).

The DIO-resistance of juvenile fish does not necessarily mean that feeding conditions do not affect adipose tissue at all. Under standard conditions, adipocytes start to be specified shortly after the initiation of external food uptake (5–7 days of age), start to be filled with lipid droplets at an age of 8 days and form white adipose tissues beginning 15 days of age [53,54]. Development of white adipose tissue has been previously shown to depend on age and size of zebrafish [54], suggesting that HF-LD fish develop adipose tissues earlier than LF-HD fish. Indeed, at 6.5 months of age, HF-LD fish had significantly more adipocytes than LF-HD fish (Fig. 3) and juvenile HF-LD fish (1.5 and 2 months of age) exhibited more adipocytes than size-matched LF-HD fish (6.5 and 18 months of age) (Fig. 6D), especially in dorsal regions (S7A Fig.). These findings indicate that young / small fish respond to increased caloric intake by increased proliferation of adipocyte progenitors, possibly preparing the adipose tissue for increased fat storage during later stages, when somatic growth rates decline.

### Middle-aged HF-LD fish display DIO and increased reproduction

Although growth rates in the (larger) HF-LD declined faster than in LF-HD fish, maximal body lengths were obtained at similar ages (12 months; Fig. 2A). This indicates that linear growth is terminated at a fixed age, irrespective of caloric intake, the actual size, and the developmental and metabolic history of the fish. Similarly, BMI values reached maximal values at an age of 9–12 months, again largely independent of the feeding conditions and the actual weight (Fig. 2C; see also below). Strikingly, the BMI dynamics were not fully reflected by TG levels (compare Fig. 2C with S3G Fig.). In NF-ND and LF-HD fish, TG levels remained rather unchanged throughout the entire period, while in HF-LD fish, maximal levels were already reached at 3 months (S3G Fig.). This suggests that between 3 and 9/12 months, rising BMI values are due to an overproportional expansion or weight-gain of other tissues, such as skeletal muscle or bones, which might increase their density. Consistently, the TG/BMI ratio of male fish progressively declined with age (Fig. 2J,K and S3K,L Fig.), suggesting that non-adipose tissues become more prominent in older fish. Indeed, TG-muscle proportions in old (18 months) males were considerably lower than in middle-aged (6.5 months) males (S3I Fig.), pointing to an increasing impact of muscle to BMI values in aging fish. Of note, however, there was a very strong linear correlation between TG levels and the BMI in HF-LD, NF-ND and LF-HD fish of the same age, making the BMI a reliable indicator of obesity and anorexia. Maximal BMI values reached were in the range of 0.8 mg/mm^2^ (0.61 mg/mm^2^ in males, 0.95 mg/mm^2^ in females), higher than values reported by Oka et al. (0.5 mg/mm^2^ for males and 0.7 mg/mm^2^ for female, obese zebrafish) [17]. This is most likely due to the different feeding regimes, as in Oka et al., fish were only overfed as adults and for 8 weeks. Compared to fish kept under standard conditions, the BMI values of our obese fish were up to two-fold increased compared to fish raised and maintained under standard conditions, while TG levels were even up to three-fold increased, thus fulfilling both criteria given by the World Health Organisation for obesity in human. Histological analyses further revealed increased numbers and sizes of adipocytes (Fig. 3), with most pronounced hypertrophy of subcutaneous adipocytes on the ventral side (belly) of the HF-LD fish (S4 Fig.), again strikingly similar to the situation in mammals. In former studies, adipocyte hypertrophy, but not adipocyte hyperplasia, was reported for obese zebrafish after transgenic overexpression of the orexic hypothalamic neuropeptide Agrp [56] or genetic loss of hypohyseal growth hormone [56,103], whereas adipocyte hyperplasia was found after transgenic overexpression of a constitutively active version of the insulin signalling transducer Akt1 [104]. This suggests that adipocyte growth and proliferation are under differential genetic control, and that hyperphagia affects both control systems. In conclusion, middle-aged fish can develop severe DIO, most likely because they cannot keep the high somatic growth rate characteristic for the DIO-resistant juvenile stages.

In addition to TG, cholesterol levels were analyzed. In contrast to obese mammals, which exhibit elevated levels of cholesterol [82,85,86], DIO zebrafish displayed lower cholesterol levels than lean control fish (Fig. 2H and S3H Fig.). Does this mean that cholesterol levels are inversely related to somatic growth, with fast-growing HF-LD fish metabolizing cholesterol more readily than slow-growing LF-HD fish? Although a tempting hypothesis, it is at least not fully supported by the shifts of cholesterol levels that occur during the entire life-time of the fish. On one hand, and in agreement with this hypothesis, aged fish that had ceased somatic growth displayed higher cholesterol levels than middle-aged fish, which still were moderately growing. However, on the other hand, maximal cholesterol levels were observed during juvenile stages, when growth rates were highest (Fig. 2H and S3H Fig.). Thus, if increased cholesterol metabolism also occurs during juvenile stages, contrary processes of cholesterol homeostasis must be predominant. In addition to its metabolism, cholesterol levels are influenced by cholesterol uptake, endogenous synthesis and excretion [65,83,84]. According to the suppliers’ information, our provided diets are low in cholesterol, making effects via differential cholesterol uptake rather unlikely. To study cholesterol synthesis, we quantified expression levels of *hmgcra*, the rate-limiting enzyme of cholesterol synthesis [87], which, however, revealed an inverse correlation between *hmgcra* mRNA and cholesterol levels. Thus, compared to fast-growing juvenile fish, *hmgcra* mRNA levels in middle-aged fish were approximately 3-fold increased (S3J Fig.), whereas cholesterol levels were half. Does this mean that the lower cholesterol levels in middle-aged fish are also not due to lower cholesterol synthesis rates? Not necessarily, as HMGCR activity could be differentially regulated at the protein level. Recently, Karanth et al. demonstrated that endogenous polyunsaturated fatty acids (PUFA) and PUFA-CoA can lower cholesterol levels through direct inhibition of HMGCR activity both in mammals and in zebrafish [105]. Such an inhibitor could be more abundant in middle-aged fish, which display higher TG levels, resulting in lower cholesterol synthesis rates and lower cholesterol levels despite the observed increased *hmgcra* expression levels. Finally, decreased cholesterol levels in middle-aged fish could be due to increased cholesterol excretion. Indeed, cholesterol elimination via fecal and biliary excretion is elevated in both normo- and hypercholesterolemic obese humans [86,106], while concomitantly enhanced cholesterol synthesis prevents hypocholesterolemia [86]. Direct cholesterol synthesis [105] and excretion assays will need to be applied in the future to distinguish between these possiblities in the zebrafish model.

Another pathway of energy expenditure in middle-aged fish is reproduction. This is of particular relevance in females, whose ovaries can account for up to one quarter of the entire body weight. Although not systematically analyzed, we found that as for scale formation (Fig. 1), HF-LD females reached sexual maturity much earlier than NF-ND or LF-HD females (at approximately 2 months compared to 4 or 5.5 months, respectively). This is similar to, but much more dramatic than in human, where menses in obese girls on average occurs one year earlier, coupled with faster somatic growth [107] and possibly mediated by Leptin, a hormone generated by adipose tissue [108]. In sexually mature HF-LD zebrafish females, relative ovarian weights were up to 2.6 fold increased compared to LF-HD siblings, largely due to enhanced oocyte growth and maturation rates, whereas total oocyte numbers remained unaltered (Fig. 5). The latter is not self-evident, as ovaries of adult zebrafish females possess oogonial nests, dividing oogonia and newly formed oocytes year round, indicative of persistent and prevalent oogonial proliferation and oogenesis beyond juvenile stages [92,109]. In mammals, both caloric restriction and obesity have a negative impact on female reproduction [110]. In genetically obese female mice, reproduction can be impaired up to complete infertility, and obese human females often display anovulatory menstrual cycling and reduced fertility [107,111,112]. Thus, while the negative effects of caloric restriction on reproduction in fish and mammals are consistent, the impact of obesity is strikingly different. We can only speculate about possible reasons. We cannot rule out that DIO in our fish was, at least in some respects, less severe than obesity in the mammalian models. However, if so, this is not reflected by the BMI values (see above) and TG levels (1.8–2.4 fold increased plasma levels in DIO zebrafish compared to 1.5–2.0 fold increases in different genetic mouse models of obesity [17,35]). Alternatively, it could be due to differences in the mode and timing of oocyte formation. In contrast to fish, all mammalian primary oocytes are formed during infancy and remain in meiotic arrest at least until puberty and without any significant growth. Thus, the process of female reproduction particularly affected in fish, oocyte growth, does not occur in mammals, while mammalian oocytes are much longer exposed to possible negative metabolic consequences of obesity.

It is also interesting to note that zebrafish HF-LD females, although requiring more energy for reproduction, were significantly longer (Fig. 2D) and had more and significantly larger adipocytes (Fig. 3) than their male siblings, raising the question about the source of the extra energy. Increased food / caloric intake would be one option, which according to our preliminary data, however, is not the case. Alternatively, compared to males, females might spend less energy in physical activity, basal metabolism or heat production. The latter point may appear far-fetched, as fish are ectotherm and lack brown adipose tissue [53], the main site of non-shivering thermogenesis in mammals. However, uncoupling of oxidative phosphorylation and thermogenesis can take place at other sites. For instance, modifications of extraocular muscles into thermogenic organs have occured in multiple teleost species, accounting for regional endothermy around the eyes and the brain [113]. Furthermore, uncoupling protein (ucp) genes are present in zebrafish and induced by low temperatures [114,115]. Future studies involving measurements of swimming activity, O_2_ consumption in resting states [116], and thermal imaging will be necessary to address these possibilities.

### Aged fish lose body fat and weight

Between an age of 9 and 12 months, all fish, irrespective of their metabolic history and actual constitution, started to lose body fat and weight. At 18 months, TG levels had dropped up to one third compared to middle-aged fish (Fig. 2 and S3 Fig.), and adipocytes had become significantly smaller (compare Fig. 3 and Fig. 4, p<0.001). In its initial phase (9–12 months), this decline was even more strongly reflected by the BMI (compare for instance Fig. 2F with Fig. 2E), as fish were still slightly growing in length (Fig. 2D). Of note, however, both linear growth termination and onset of fat wasting were strictly linked to the age of the fish. We do not know the mechanisms underlying this age-dependent regulation. Gradual loss of telomerase activity, resulting in telomere shortening and chromosomal instabiliy, as formerly proposed as a functional link between fish aging and determinate growth [117], seems unlikely, as somatic zebrafish tissues display unaltered telomerase activity from embryos through aged adults [118].

Also in human, fat tissue mass increases through middle ages and declines in advanced ages, when adipocytes display an impaired ability to accumulate lipid [119–121], consistent with the reduced adipocyte sizes in aged zebrafish (Fig. 4). In addition, elderly humans display compromised adipocyte renewal due to dysdifferentiation of adipocyte progenitors into a pro-inflammatory, tissue-remodeling, senescent-like state [122]. Of note, however, no such adipocyte hypoplasia was observed in our aged zebrafish (Fig. 4).

Another factor contributing to fat tissue and weight loss in elderly humans is reduced appetite and undernutrition [95,96]. Normally, Leptin, a hormone released by adipose tissue, signals to the hypothalamic control center of energy homeostasis, restricting appetite and food uptake (see also below). Accordingly, the reduction of adipose tissue and leptin plasma levels in elderly should cause increased appetite, the exact opposite of what is observed, pointing to disturbances in this negative feedback loop or other regulatory pathways. Future experiments, including quantification of food intake and comparative analyses of the neuroendocrine control system will be necessary to investigate whether similar misregulations are at play in aged zebrafish.

### The sense of differential energy allocation

Comparing the different caloric intake-dependent processes with each other, we noted differential energy allocation in response to caloric restriction. During juvenile stages, scale formation, and most likely other processes of metamorphosis as well [68], were prioritized over somatic growth and occurred at smaller body sizes (Fig. 6A-C,K), similar to recently reported findings in zebrafish mutants lacking pituitary growth hormone [103]. In addition, in middle-aged fish, somatic growth and reproduction were priorized over fat storage (Fig. 6D-G,J,K). In sexually mature females, we even observed size-dependent switches in differential energy allocation into somatic versus ovarian growth. In starving small females, somatic growth was slightly priorized over ovarian growth, whereas it was vice versa in starving larger females (Fig. 6H,I,K).

These differential allocations make sense, as they increase the likelihood that individuals will reach their ultimate goal, reproduction and survival of the species beyond the current generation. Teleost metamorphosis involves the development of new adult features and major remodelings of different organ systems that are crucial for a life as sexually mature adults [122]. It is temporally and—most likely—also mechanistically linked with sexual differentiation of the larval hermaphrodites to males or females [122,123]. In this light, priorization of metamorphosis upon caloric restriction will assure the development of adult features and reproducibility even when fish are of smaller size [68]. On the other hand, reproduction in females is biased by the size of mature oocytes, which are about 0.7 mm in diameter, independently of the feeding conditions and actual sizes of the females. Thus, a certain somatic size is necessary to allow proper oocyte accommodation and spawning, explaining why small females initially have to priorize somatic over ovarian growth. When larger and meeting these physical conditions, however, relatively more energy is invested into oocyte growth and the production of offspring, which due to their different diet demands might be less affected by the nutrient shortage. The latter response is obviously different from mammals, in which juvenile undernutrition has more dramatic effects on female fecundity than somatic growth [124], which makes sense as compared to fish, mammalian females have to invest much more caloric energy per offspring.

### How is differential energy allocation regulated on the neuroendocrine level?

The balance between energy uptake, storage and expenditure is under neuroendocrine control, with a central role of the melanocortin system located in the hypothalamus of the brain [125–127]. First-order neurons of the melanocortin system sense the energy status of the body via metabolites like plasma glucose and hormones like leptin from adipose tissue and insulin from the pancreas [128–131]. In response to these cues, they express either proopiomelanocortin (POMC) or Agouti-related peptide (AGRP), which in turn signal to second-order neurons [128,129,131], characterized by the expression of MC3R and/or MC4R, members of the melanocortin receptor family of G-protein coupled receptors [128,132,133]. alpha-melanocyte stimulating hormone (α-MSH), a product of POMC [134,135], is the endogenous agonist and agouti-related peptide (AGRP) the inverse agonist of MC3R and MC4R [128,129]. MC4R signaling in the different second-order neurons has anorexic effects, leading to decreased food intake and increased energy expenditure by influencing the different pathways of energy expenditure. In mammals, two types of second-order neurons have been well characterized, both of which are hypophysiotropic and regulate pituitary hormone release [125,127]. These are: (1) thyrotropin-releasing hormone (TRH) neurons, which regulate basal metabolism and body growth via the adenohypophyseal hormone TSH and thyroid hormones of the thyroid gland [136–140]. (2) corticotropin-releasing hormone (CRH) neurons, which regulate stress-induced physical activity and food intake [65,141–144]. In addition, other hypothamalmic and hypophysiotropic neurons may be under direct control of the melanocortin system, such as (3) gonadotropin-releasing hormone (GnRH) neurons, which regulate reproduction [145–149], and (4) growth hormone releasing hormone (GHRH) neurons in the ARC and somatostatin (SST) neurons in the PVA, which regulate somatic body growth via stimulation (GHRH) or inhibition (SST) of growth hormone (GH) release by the pituitary [60,150–154].

In zebrafish, *pomc* and *agrp* neurons are present in corresponding regions of the hypothalamus, fulfilling similar functions. Thus, *agrp* expression is stimulated upon caloric restriction [59], while transgenic overexpression of *agrp* [56] and genetic ablation of *mc4r* [60] lead to obesity and increased somatic growth. Furthermore, likely second-order neurons such as *crh*, *trh*, *gnrh*, *ghrh* and *sst*-positive neurons are present in corresponding hypothalamic nuclei ([51] and references therein). Future studies of these neural circuits, including calcium imaging of neuronal activity in the different potential second-order neurons upon caloric restriction and hyperphagia, will be necessary to unravel the cellular and neuroendocrine basis of the differential energy allocations observed in this work. Furthermore, comparative analyses between zebrafish and mouse will help to eludicate the neuroendocrine basis of the conserved and different responses in teleosts and mammals. For example, a tighter integration of *ghrh* and *sst* neurons into the melanocortin system could explain the more pronounced effects of caloric intake on somatic growth in fish, while differences in the regulation of *gnrh* neurons could underlie the prioritization of reproduction in middle-aged zebrafish females. Work described here has prepared the ground for such future functional studies, helping to better link genetic, molecular and cellular regulators of energy homeostasis to the different pathways of energy expenditure, and facilitating comparisons between fish and mammals.

## Supporting Information

S1 Fig Validation of technique used to determine mean adipocyte sizes. Comparison of mean adipocyte size value calculated from measured single adipocyte cell sizes (left column) with the mean value obtained by dividing the total area of subcutaneous adipocyte tissue by the total number of adipocytes (right column). The same H&E-stained longitudinal sections through a HF-LD fish of 6.5 months of age were used. Obtained values are very similar (approximately 3000 μm^2^). In the left column mean +/- standard deviation is indicated. Please note that the distribution of individual adipocyte sizes is rather continuous, displaying no signs of a bimodal pattern [91].
(TIF)

S2 Fig Somatic growth of larval and juvenile fish depends on husbandry conditions. Growth curves of fish kept at ad libidum feeding conditions (HF-LD, black rhombuses), in comparison to fish kept at 10x higher density, but receiving the same amount of food per tank as the HF fish (LF-HD, white squares), and in comparison to fish kept at the same density as the HF fish, but receiving only 10% of the food (LF-LD; white triangles), n = 10 for each condition. For detailed description of feeding regimes, see Materials and Methods.
(TIF)

S3 Fig Hyperphagia results in increased body length and weight, increased BMI, increased triglyceride, but descreased cholesterol levels—additions to Fig. 2. A-F: Body length (A,D), body weight (B,E) and BMI (C,F) curves of male and female NF-ND (A-C) and LF-HD (D-F) fish between 3 and 18 months of age, * indicates significant differences with p<0.05 and ** with p<0.01 according to ANOVA followed by the Least Significant Difference (Bonferroni’s) test, n ≥ 5; similar results were obtained in two additional independent experiments. G,H: Curves of whole body levels of triglyceride (TG) (G) and cholesterol (H) of LF-HD, NF-ND and HF-LD fish between 1 month and 18 months of age, pools of fish were analysed for 1 month (10 (HF-LD), 25 (NF-ND) or 50 fish (LF-HD), n = 2–6) and 3 months old fish (3 (HF-LD) or 5 fish (NF-ND, LF-HD), n = 3–4), while for older ages 5 male individuals per condition were analysed (n = 5), * indicates significant differences with p<0.05 and *** p<0.001 according to ANOVA followed by the Least Significant Difference (Bonferroni’s) test, n = 5; similar results were obtained in a second, independent experiment. I: Proportion of skeletal muscular area in % body (without swimming bladder and internal organs) of male HF-LD und LF-HD fish of indicated ages, n.s.: not significant according to ANOVA followed by the Least Significant Difference (Bonferroni’s) test; n = 4 (2 males per condition and age and 2 analysed section per male); J: Quantitative real-time PCR showing relative expression of *hmgcra*, encoding the rate-limiting enzyme of cholesterol synthesis, in young (1 month) DIO-resistant and middle-aged (7 months) DIO male zebrafish, *** indicates significant differences (p<0.001) according to the Student`s t-test; n = 3. K-L: Correlation of BMI and whole body TG for male LF-HD and HF-LD fish at 9 months (K) and 12 months (L) of age.
(TIF)

S4 Fig Obesity in 6.5 months old HF-LD fish is accompanied with differential and region-specific hyperplasia and hypertrophy of subcutaneous adipocytes, while keratinocytes are largely unaffected. A: Schematic overview of the different dorsoventral levels (1–5) at which longitudinal sections were used to analyze subcutaneous adipocytes; B: overview of an H&E stained section of a male HF-LD fish at level 2; boxes show locations of areas shown in Fig. 3 A-H. C,D: Numbers of subcutaneous adipocytes per level (C) and sizes (D) of subcutaneous adipocytes of male and female LF-HD and HF-LD fish in each of the 5 analyzed levels, n = 8 (2 fish per condition, 2 sections per level and 2 sides per section); E,F: Thickness of the skin (E) and sizes of keratinocytes (F) of male and female LF-HD and HF-LD fish, determined at the level of the most posterior scale for each level, means of all levels are shown; columns with same superscript letter are not significantly different (p>0.05) according to ANOVA followed by the Least Significant Difference (Bonferroni’s) test, n = 20 (for thickness, 2 fish per condition, 5 measurements per side of the analysed section) or n = 40 (for size, 2 fish per condition, 10 measured keratinocytes per side of the analysed section). Similar results were obtained in second, independent experiments.
(TIF)

S5 Fig Reduction of BMI and TG levels in 18 months old male zebrafish is accompanied with a reduction in adipocyte sizes. A: Schematic overview of the different levels of which longitudinal sections were used to analyze subcutaneous adipocytes; B: overview of an H&E stained section of a male HF-LD fish at level 2, boxes show locations of the areas shown in Fig. 4 A-H; C-D: Numbers (C) and sizes (D) of subcutaneous adipocytes of male LF-HD and HF-LD fish with an age of 18 months shown for each of the 5 analyzed levels, n = 8 (2 fish per condition, 2 sections per level and 2 sides per section); E,F: Thickness of the skin (E) and sizes of keratinocytes (F) of male LF-HD and HF-LD fish, determined at the level of the most posterior scale for each level, means of all levels are shown; *** indicate significant difference with p<0.001 according to the Student`s T test, n = 20 (for thickness, 2 fish per condition, 5 measurements per side of the analysed section) or n = 40 (for size, 2 fish per condition, 10 measured keratinocytes per side of the analysed section). Similar results were obtained in second, independent experiments.
(TIF)

S6 Fig Caloric intake affects ovarian sizes and oocyte growth rates in in 8.5 months old females—additions to Fig. 5. A: Schematic overview of the different levels at which longitudinal sections were used to analyze oocytes. B: Overview of an H&E stained section of a female HF-LD fish at level 4; boxes show location of the area shown in Fig. 5E,F. C: Numbers of oocytes of LF-HD and HF-LD females in each of the 4 analyzed levels, n = 3 for level 1–4, n = 12 for all levels. D: Absolute numbers of oocytes per maturation stage of HF-LD and LF-HD females in all of the four analyzed dorsoventral levels; * indicates a significant difference (p<0.05), n.s.means not significant according to the Student`s T test, n = 3. Similar results were obtained in a second, independent experiment.
(TIF)

S7 Fig Size-matched HF-LD fish exhibit differential and region-specific hyperplasia and hypertrophy of subcutaneous adipocytes compared to LF-HD males. A,B: Comparison of numbers (A) and sizes (B) of subcutaneous adipocytes of middle-aged (left side of each panel) and aged (right side of each panel) LF-HD males (white columns) with size-matched HF-LD males (striped columns) and with age-matched HF-LD male siblings (black columns). Standard lengths and ages of analyzed fish were: middle-aged LF-HD: 21.2 +/- 1.5 mm, 6.5 months; sized-matched HF-LD: 20.8 +/- 1.1 mm, 1.5 months; age-matched HF-LD: 28.8 +/- 0.9 mm, 6.5 months; aged LF-HD: 22.5 +/- 1.0 mm, 18 months; size-matched HF-LD: 23.0 +/- 0,8 mm, 2 months; age-matched HF-LD: 32.3 +/- 1.0 mm, 18 months. Values are separately shown for each of the investigated 5 levels along the dorsoventral axis of the fish (1 = dorsal-most, 5 = ventral-most; compare with S4 Fig.). Stars indicate significant differences with p<0.05 (*), p<0.01 (**), or p<0.001 (***), and n.s. means not significant, according to ANOVA followed by the Least Significant Difference (Bonferroni’s) test; n = 8 (2 fish per condition, 2 sections per level and 2 sides per section).
(TIF)
