# Supplementary figures and images for: Long-Term Hyperphagia and Caloric Restriction Caused by Low- or High-Density Husbandry Have Differential Effects on Zebrafish Postembryonic Development, Somatic Growth, Fat Accumulation and Reproduction
Source: PLoS One. 2015 Mar 23;10(3):e0120776. doi: 10.1371/journal.pone.0120776 (PMC4370574; doi:10.1371/journal.pone.0120776)

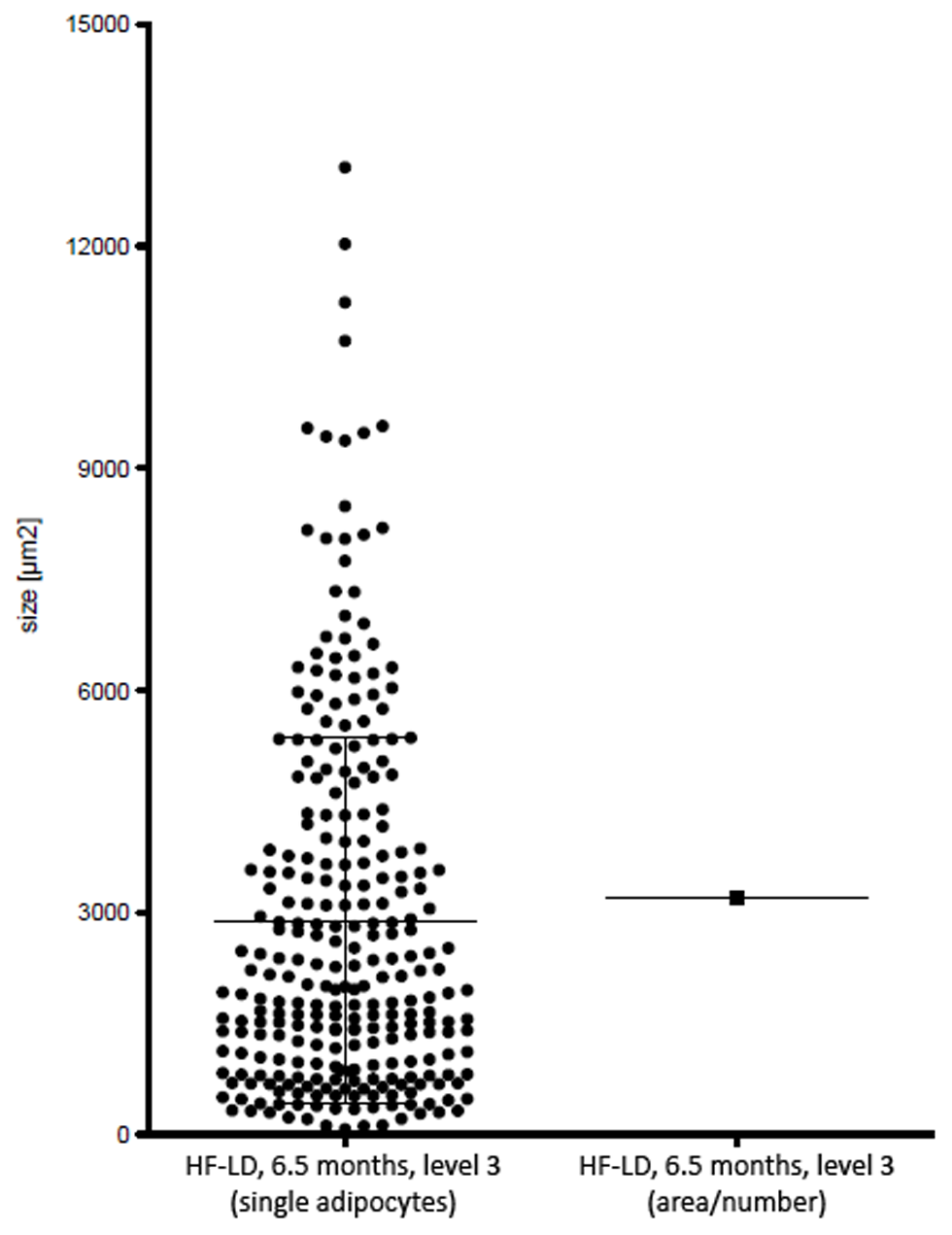

Supplement: S1 Fig — Comparison of mean adipocyte size value calculated from measured single adipocyte cell sizes (left column) with the mean value obtained by dividing the total area of subcutaneous adipocyte tissue by the total number of adipocytes (right column). The same H&E-stained longitudinal sections through a HF-LD fish of 6.5 months of age were used. Obtained values are very similar (approximately 3000 μm2). In the left column mean +/- standard deviation is indicated. Please note that the distribution of individual adipocyte sizes is rather continuous, displaying no signs of a bimodal pattern [91]. (TIF) [file pone.0120776.s001.tif]

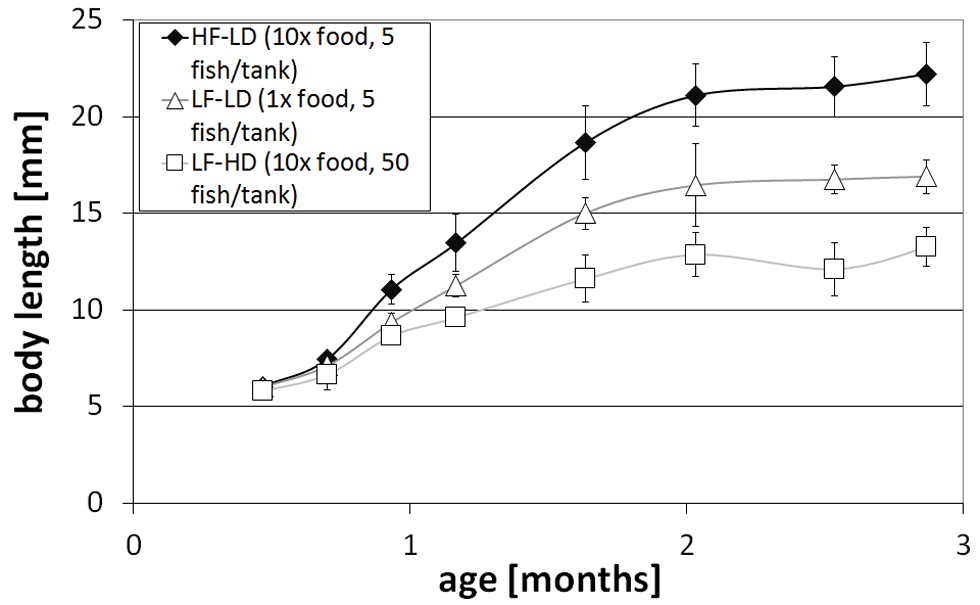

Supplement: S2 Fig — Growth curves of fish kept at ad libidum feeding conditions (HF-LD, black rhombuses), in comparison to fish kept at 10x higher density, but receiving the same amount of food per tank as the HF fish (LF-HD, white squares), and in comparison to fish kept at the same density as the HF fish, but receiving only 10% of the food (LF-LD; white triangles), n = 10 for each condition. For detailed description of feeding regimes, see Materials and Methods. (TIF) [file pone.0120776.s002.tif]

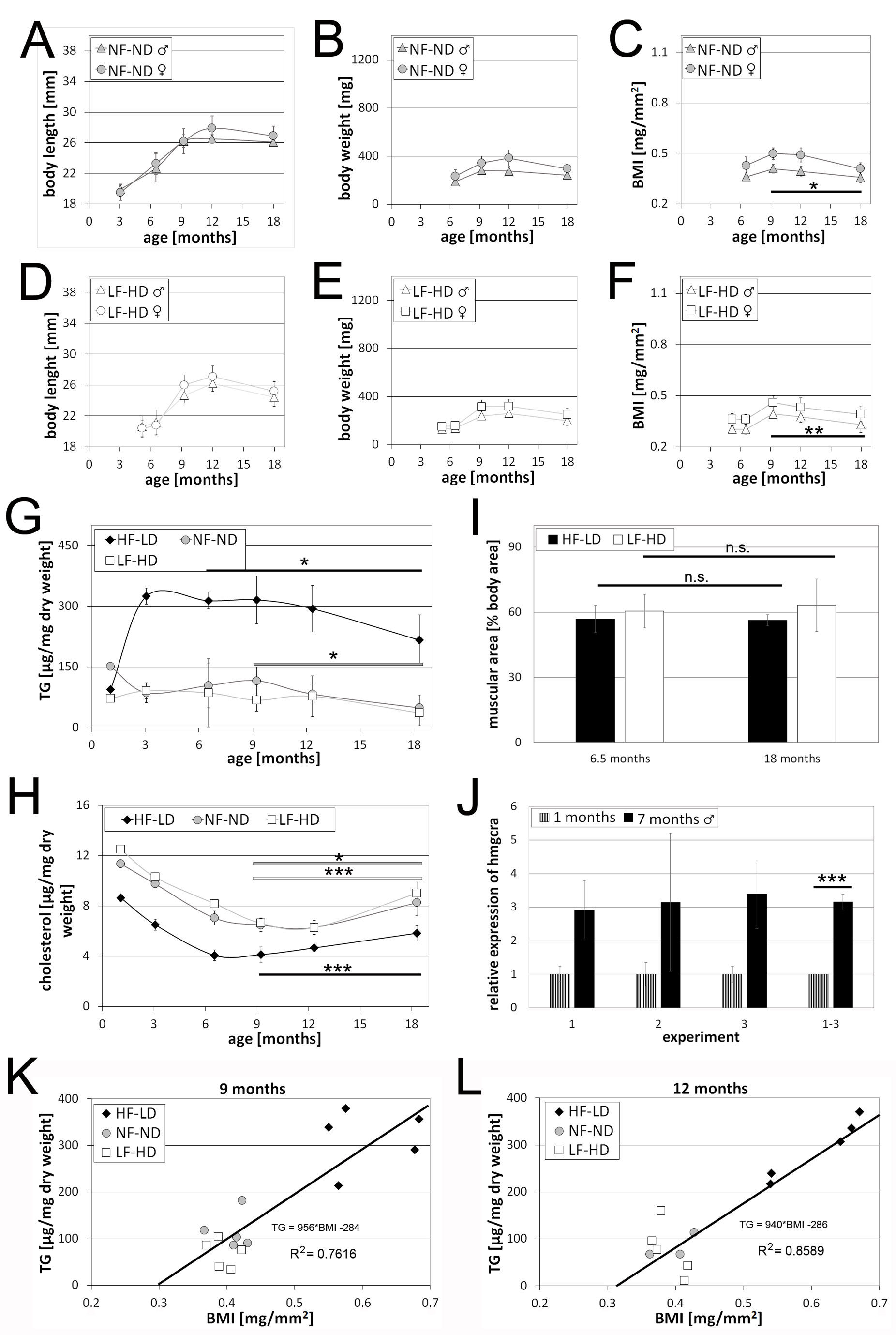

Supplement: S3 Fig — A-F: Body length (A,D), body weight (B,E) and BMI (C,F) curves of male and female NF-ND (A-C) and LF-HD (D-F) fish between 3 and 18 months of age, * indicates significant differences with p<0.05 and ** with p<0.01 according to ANOVA followed by the Least Significant Difference (Bonferroni’s) test, n ≥ 5; similar results were obtained in two additional independent experiments. G,H: Curves of whole body levels of triglyceride (TG) (G) and cholesterol (H) of LF-HD, NF-ND and HF-LD fish between 1 month and 18 months of age, pools of fish were analysed for 1 month (10 (HF-LD), 25 (NF-ND) or 50 fish (LF-HD), n = 2–6) and 3 months old fish (3 (HF-LD) or 5 fish (NF-ND, LF-HD), n = 3–4), while for older ages 5 male individuals per condition were analysed (n = 5), * indicates significant differences with p<0.05 and *** p<0.001 according to ANOVA followed by the Least Significant Difference (Bonferroni’s) test, n = 5; similar results were obtained in a second, independent experiment. I: Proportion of skeletal muscular area in % body (without swimming bladder and internal organs) of male HF-LD und LF-HD fish of indicated ages, n.s.: not significant according to ANOVA followed by the Least Significant Difference (Bonferroni’s) test; n = 4 (2 males per condition and age and 2 analysed section per male); J: Quantitative real-time PCR showing relative expression of hmgcra, encoding the rate-limiting enzyme of cholesterol synthesis, in young (1 month) DIO-resistant and middle-aged (7 months) DIO male zebrafish, *** indicates significant differences (p<0.001) according to the Student`s t-test; n = 3. K-L: Correlation of BMI and whole body TG for male LF-HD and HF-LD fish at 9 months (K) and 12 months (L) of age. (TIF) [file pone.0120776.s003.tif]

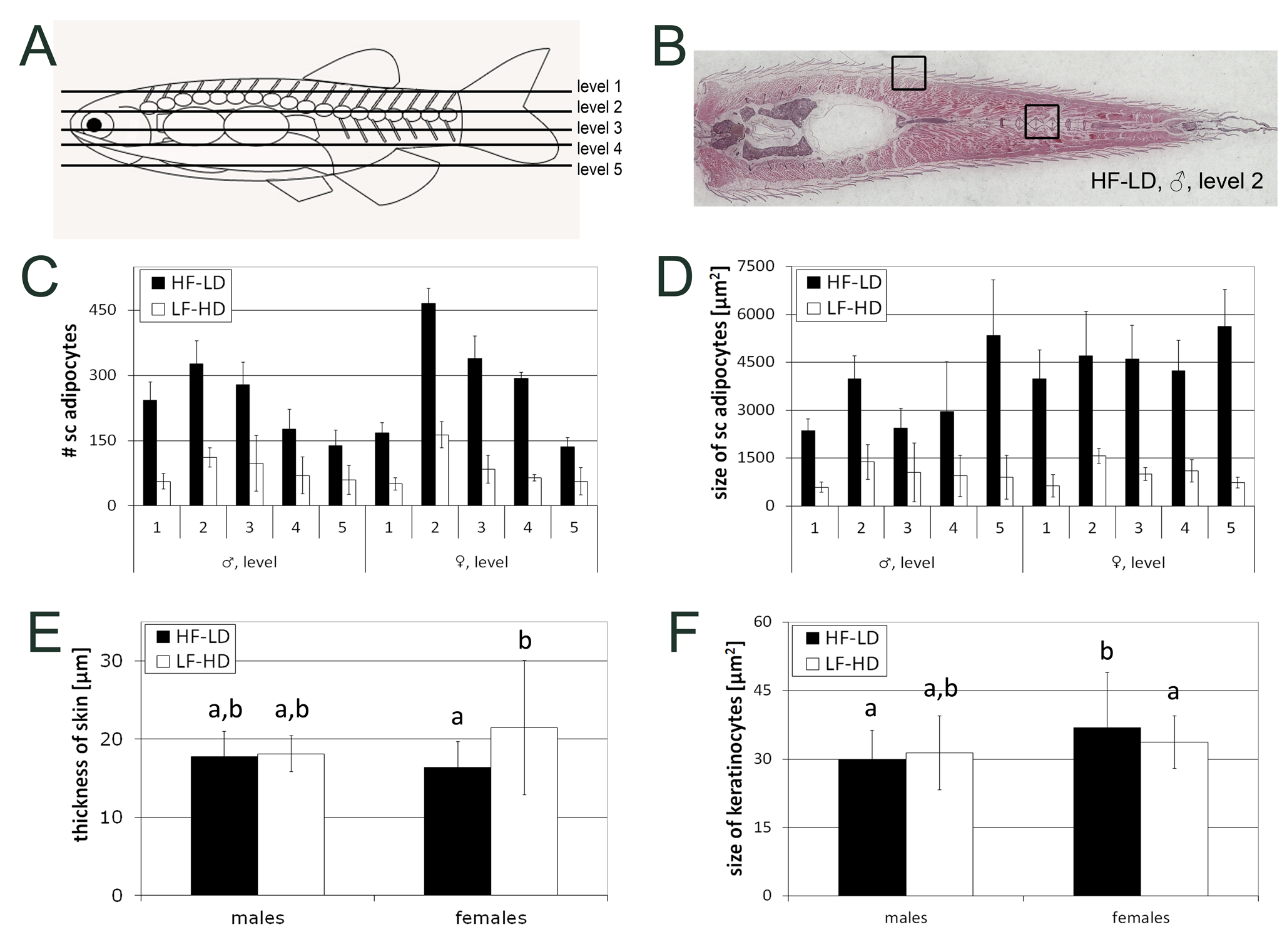

Supplement: S4 Fig — A: Schematic overview of the different dorsoventral levels (1–5) at which longitudinal sections were used to analyze subcutaneous adipocytes; B: overview of an H&E stained section of a male HF-LD fish at level 2; boxes show locations of areas shown in Fig. 3 A-H. C,D: Numbers of subcutaneous adipocytes per level (C) and sizes (D) of subcutaneous adipocytes of male and female LF-HD and HF-LD fish in each of the 5 analyzed levels, n = 8 (2 fish per condition, 2 sections per level and 2 sides per section); E,F: Thickness of the skin (E) and sizes of keratinocytes (F) of male and female LF-HD and HF-LD fish, determined at the level of the most posterior scale for each level, means of all levels are shown; columns with same superscript letter are not significantly different (p>0.05) according to ANOVA followed by the Least Significant Difference (Bonferroni’s) test, n = 20 (for thickness, 2 fish per condition, 5 measurements per side of the analysed section) or n = 40 (for size, 2 fish per condition, 10 measured keratinocytes per side of the analysed section). Similar results were obtained in second, independent experiments. (TIF) [file pone.0120776.s004.tif]

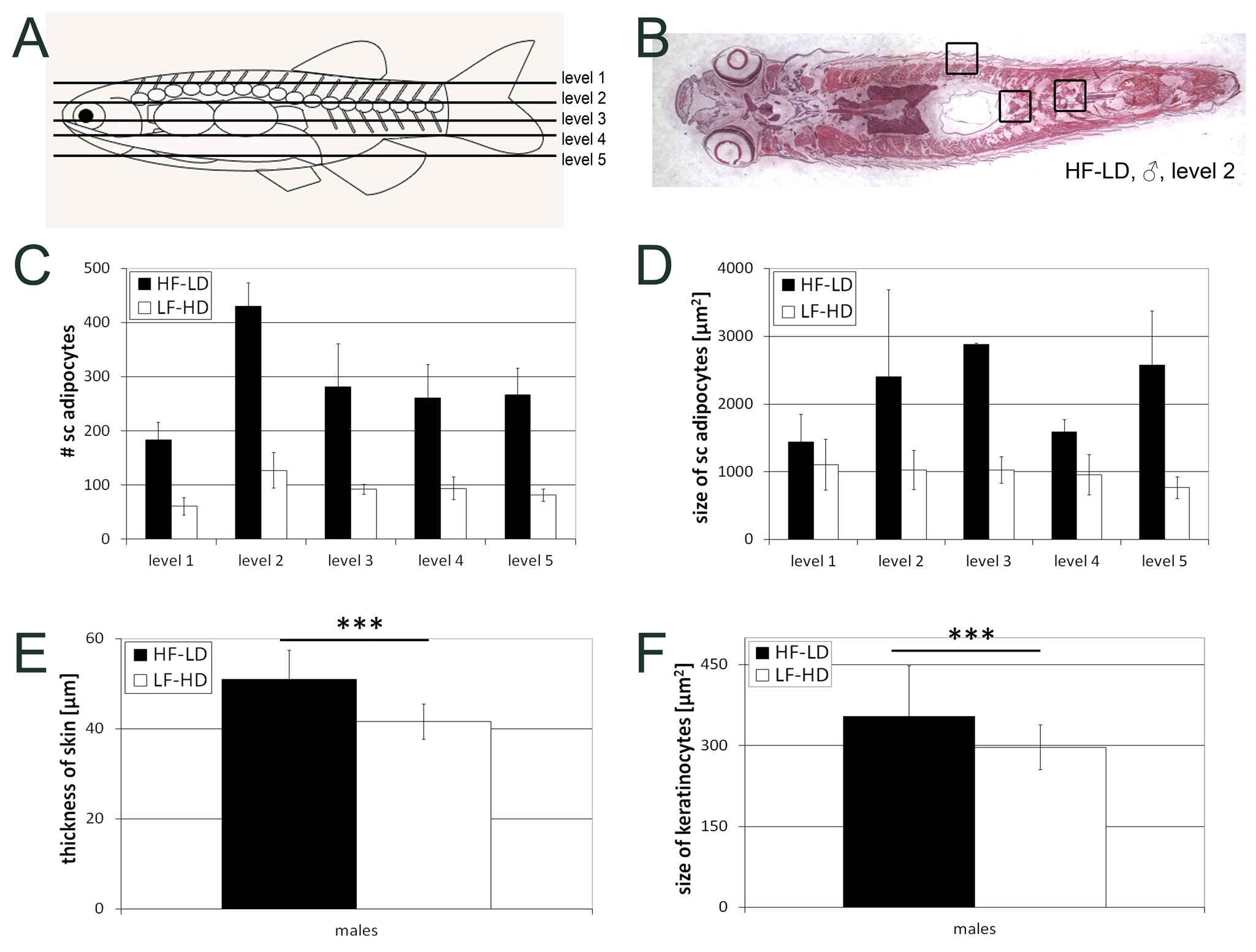

Supplement: S5 Fig — A: Schematic overview of the different levels of which longitudinal sections were used to analyze subcutaneous adipocytes; B: overview of an H&E stained section of a male HF-LD fish at level 2, boxes show locations of the areas shown in Fig. 4 A-H; C-D: Numbers (C) and sizes (D) of subcutaneous adipocytes of male LF-HD and HF-LD fish with an age of 18 months shown for each of the 5 analyzed levels, n = 8 (2 fish per condition, 2 sections per level and 2 sides per section); E,F: Thickness of the skin (E) and sizes of keratinocytes (F) of male LF-HD and HF-LD fish, determined at the level of the most posterior scale for each level, means of all levels are shown; *** indicate significant difference with p<0.001 according to the Student`s T test, n = 20 (for thickness, 2 fish per condition, 5 measurements per side of the analysed section) or n = 40 (for size, 2 fish per condition, 10 measured keratinocytes per side of the analysed section). Similar results were obtained in second, independent experiments. (TIF) [file pone.0120776.s005.tif]

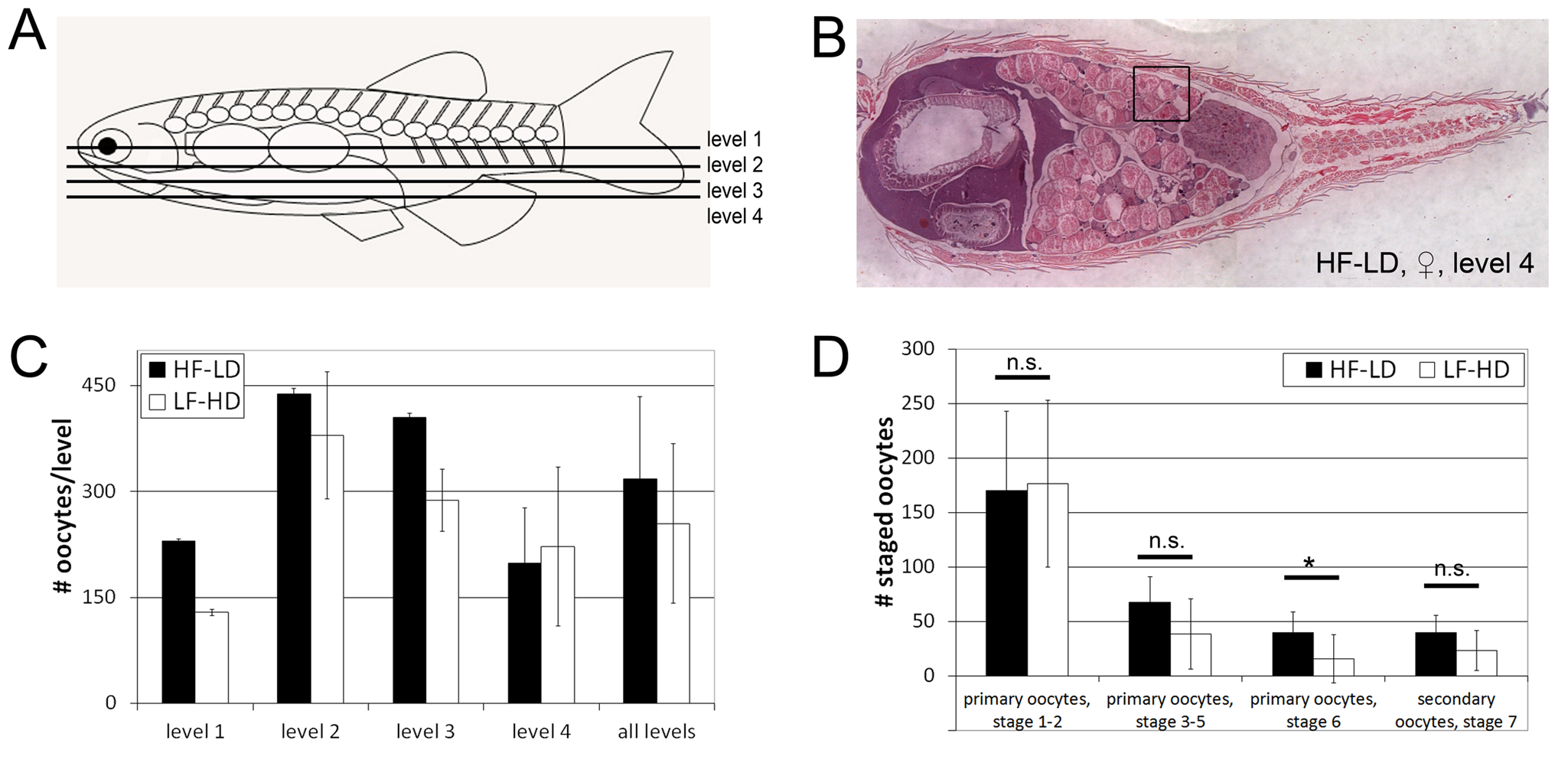

Supplement: S6 Fig — A: Schematic overview of the different levels at which longitudinal sections were used to analyze oocytes. B: Overview of an H&E stained section of a female HF-LD fish at level 4; boxes show location of the area shown in Fig. 5E,F. C: Numbers of oocytes of LF-HD and HF-LD females in each of the 4 analyzed levels, n = 3 for level 1–4, n = 12 for all levels. D: Absolute numbers of oocytes per maturation stage of HF-LD and LF-HD females in all of the four analyzed dorsoventral levels; * indicates a significant difference (p<0.05), n.s.means not significant according to the Student`s T test, n = 3. Similar results were obtained in a second, independent experiment. (TIF) [file pone.0120776.s006.tif]

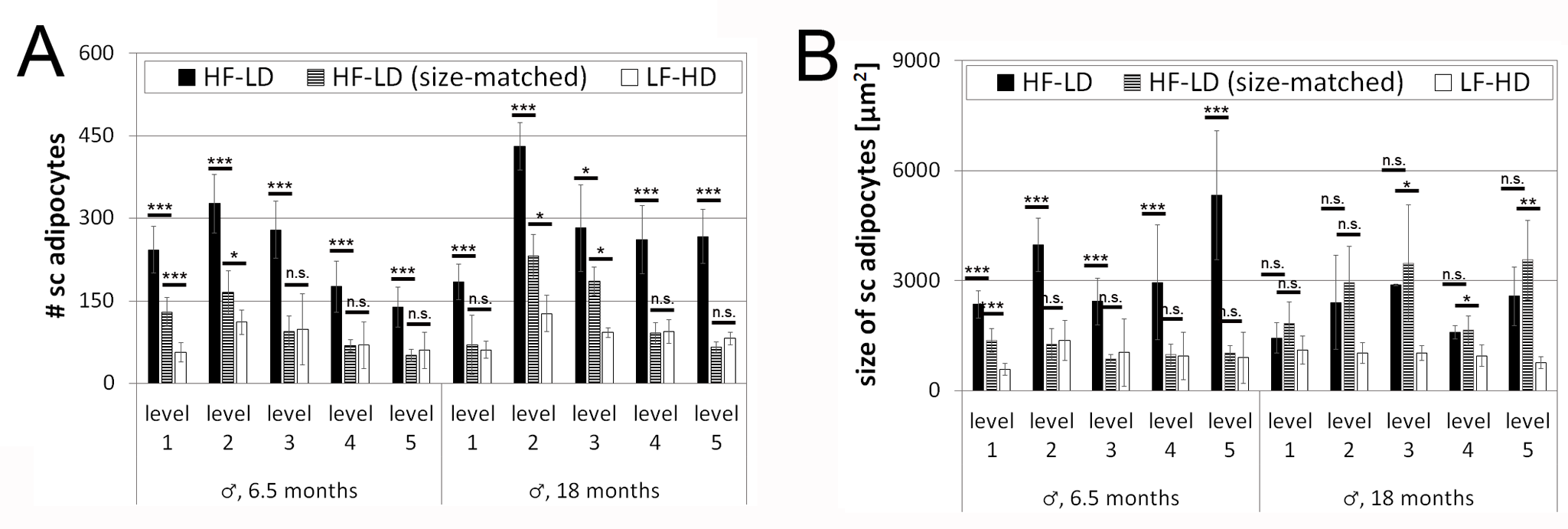

Supplement: S7 Fig — A,B: Comparison of numbers (A) and sizes (B) of subcutaneous adipocytes of middle-aged (left side of each panel) and aged (right side of each panel) LF-HD males (white columns) with size-matched HF-LD males (striped columns) and with age-matched HF-LD male siblings (black columns). Standard lengths and ages of analyzed fish were: middle-aged LF-HD: 21.2 +/- 1.5 mm, 6.5 months; sized-matched HF-LD: 20.8 +/- 1.1 mm, 1.5 months; age-matched HF-LD: 28.8 +/- 0.9 mm, 6.5 months; aged LF-HD: 22.5 +/- 1.0 mm, 18 months; size-matched HF-LD: 23.0 +/- 0,8 mm, 2 months; age-matched HF-LD: 32.3 +/- 1.0 mm, 18 months. Values are separately shown for each of the investigated 5 levels along the dorsoventral axis of the fish (1 = dorsal-most, 5 = ventral-most; compare with S4 Fig.). Stars indicate significant differences with p<0.05 (*), p<0.01 (**), or p<0.001 (***), and n.s. means not significant, according to ANOVA followed by the Least Significant Difference (Bonferroni’s) test; n = 8 (2 fish per condition, 2 sections per level and 2 sides per section). (TIF) [file pone.0120776.s007.tif]
